# Supplementary material for: A human-specific non-coding RNA for EFHC1, an epilepsy-associated gene, regulates neural stem cell proliferation for cortical development
Source: Stem Cell Reports. 2026 Aug 20;21(9):103049. doi: 10.1016/j.stemcr.2026.103049 (PMC13555433; doi:10.1016/j.stemcr.2026.103049)
Supplement: Supplementary file 1 [file mmc1.pdf]

Stem Cell Reports, Volume *21*

Supplemental Information

A human-specific non-coding RNA for *EFHC1*, an epilepsy-associated gene, regulates neural stem cell proliferation for cortical development

Sota Nishida, Boyang An, Hayato Uchida, Shota Zenno, Takumi Nojima, Arisa Makimura, Fumihiro Morishita, Mizuki Honda, and Takuya Imamura

Fig.S1

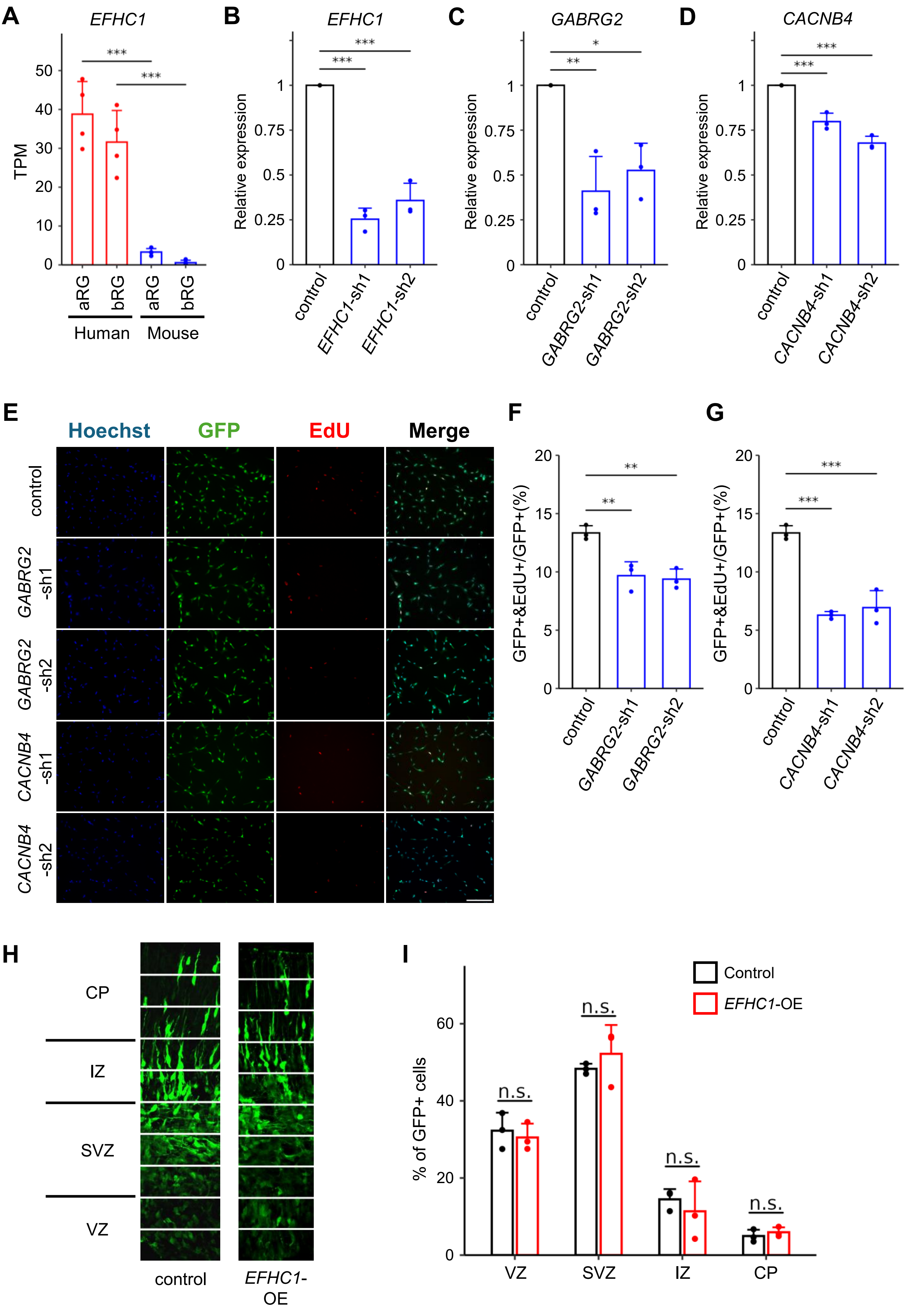
A
EFHC1
TPM
aRG
bRG
aRG
bRG
Human
Mouse
B
EFHC1
Relative expression
control
EFHC1-sh1
EFHC1-sh2
C
GABRG2
Relative expression
control
GABRG2-sh1
GABRG2-sh2
D
CACNB4
Relative expression
control
CACNB4-sh1
CACNB4-sh2
E
Hoechst
GFP
EdU
Merge
control
GABRG2 -sh1
GABRG2 -sh2
CACNB4 -sh1
CACNB4 -sh2
F
GFP+&EdU+/GFP+(%)
control
GABRG2-sh1
GABRG2-sh2
G
GFP+&EdU+/GFP+(%)
control
CACNB4-sh1
CACNB4-sh2
H
CP
IZ
SVZ
VZ
control
EFHC1-OE
I
Control
EFHC1-OE
% of GFP+ cells
n.s.
VZ
SVZ
IZ
CP

Fig. S1

JME-associated genes that are highly expressed in humans play a role in NSC proliferation.

(A) The expression levels of *EFHC1* in aRG and bRG of humans and mice. $n = 4$ independent experiments; Tukey's test.

(B-D) Quantification of the mRNA level of *EFHC1* (B), *GABRG2* (C), and *CACNB4* (D) following knockdown. $n = 3$ independent experiments; Tukey's test.

(E) Representative images of human NSCs stained for GFP (green) and EdU (red) in control, *GABRG2* knockdown, and *CACNB4* knockdown. Nuclei were stained with Hoechst (blue).

(F and G) Quantification of EdU+ GFP+ cells to GFP+ cells under *GABRG2* knockdown (F) and *CACNB4* knockdown (G) as shown in (E). $n = 3$ independent experiments; Tukey's test.

(H) Representative images of the cortical slices stained for GFP (green) in control and *EFHC1*-overexpressing E15.5 developing mice. These images show the spatial distribution of GFP-positive cells across cortical developmental zones.

(I) Quantification of the ratio of GFP+ cells in the VZ, SVZ, IZ, and CP, respectively. n = 3 independent experiments; Tukey's test.

mean ± SD; *$P < 0.05$, **$P < 0.01$, ***$P < 0.001$; scale bar = 100 µm.

Fig.S2

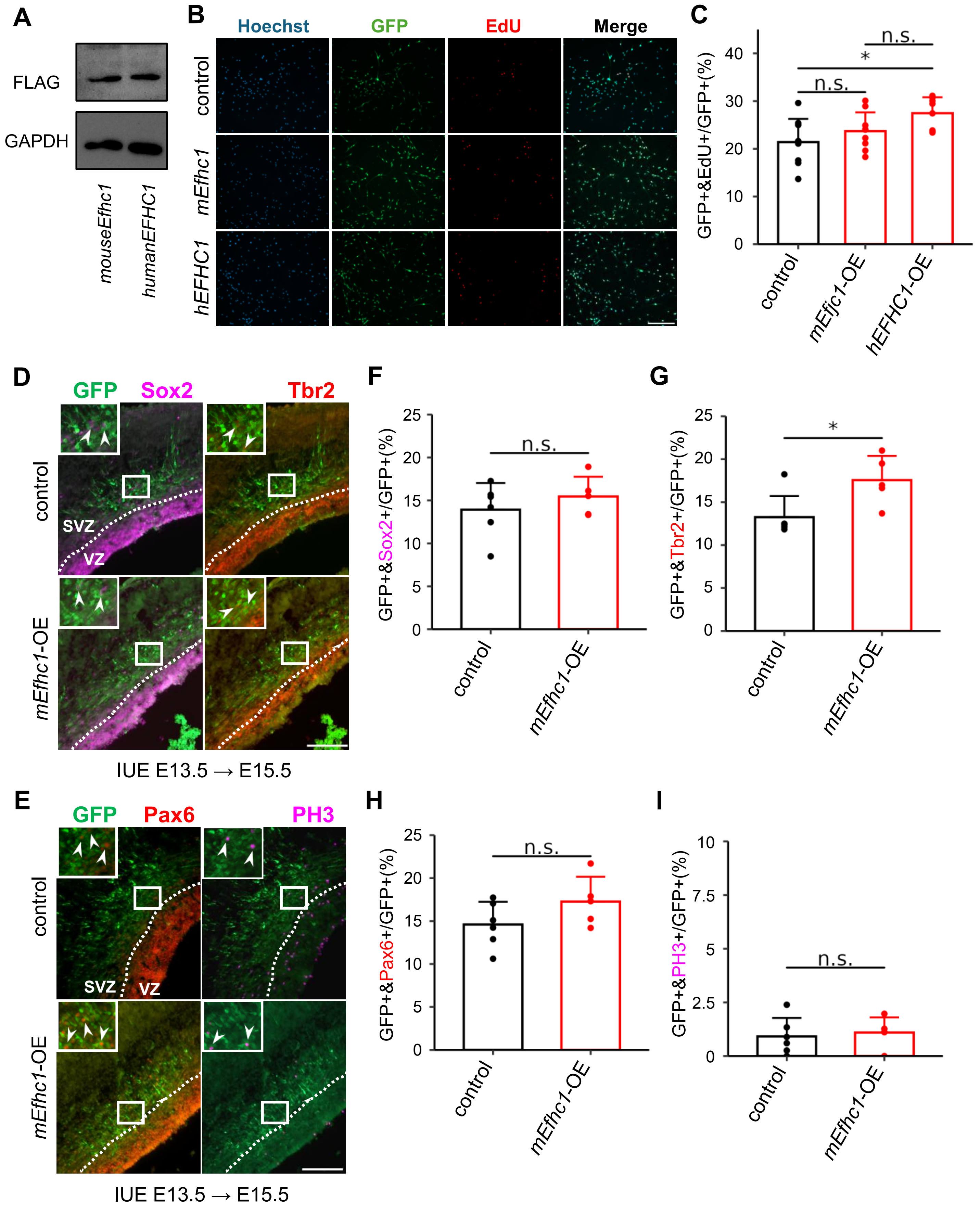
A
FLAG
GAPDH
mouseEfhc1
humanEFHC1
B
Hoechst
GFP
EdU
Merge
control
mEfhc1
hEFHC1
C
GFP+&EdU+/GFP+(%)
40
30
20
10
0
n.s.
*
n.s.
control
mEfjc1-OE
hEFHC1-OE
D
GFP
Sox2
Tbr2
control
mEfhc1-OE
SVZ
VZ
IUE E13.5 → E15.5
F
GFP+&Sox2+/GFP+(%)
25
20
15
10
5
0
n.s.
control
mEfhc1-OE
G
GFP+&Tbr2+/GFP+(%)
25
20
15
10
5
0
*
control
mEfhc1-OE
E
GFP
Pax6
PH3
control
mEfhc1-OE
SVZ
VZ
IUE E13.5 → E15.5
H
GFP+&Pax6+/GFP+(%)
25
20
15
10
5
0
n.s.
control
mEfhc1-OE
I
GFP+&PH3+/GFP+(%)
10
7.5
5
2.5
0
n.s.
control
mEfhc1-OE

Fig. S2

Mouse *Efhc1* expression slightly promotes NSC proliferation.

(A) Western blot analysis of whole cell lysates from mouse NSCs overexpressing either *mouseEfhc1* or *humanEFHC1*. The expression levels of *mouseEfhc1* and *humanEFHC1* were compared using the FLAG tag present in both vectors.

(B) Representative images of E14.5 mouse NSCs stained for GFP (green) and EdU (red) in control, human *EFHC1* overexpression, and mouse *Efhc1* overexpression. Nuclei were stained with Hoechst (blue).

(C) Quantification of the ratio of EdU+ GFP+ cells to GFP+ cells shown in (A). $n = 9$ (control and *mEfhc1*-OE) and 8 (*hEFHC1*-OE) independent experiments; Tukey's test.

(D) Representative images of the cortical slices stained for GFP (green), Sox2 (magenta), and Tbr2 (red) in control and mouse *Efhc1*-overexpressing E15.5 developing mice. Nuclei were stained with Hoechst (blue).

(E) Representative images of the cortical slices stained for GFP (green), Pax6 (red), and PH3 (magenta) in control and mouse *Efhc1*-overexpressing E15.5 developing mice. Nuclei were stained with Hoechst (blue).

(F-I) Quantification of the ratio of Sox2+ (E), Tbr2+ (F), Pax6+ (G), and PH3+ (H) GFP+ cells to GFP+ cells in SVZ. $n = 6$ (control) and 5 (*mEfhc1*-OE) independent experiments; Student's *t* test.

IUE: *in utero* electroporation; OE: overexpression; mean ± SD; $^*P < 0.05$, n.s.: not significant; scale bar = 100 μm.

Fig.S3

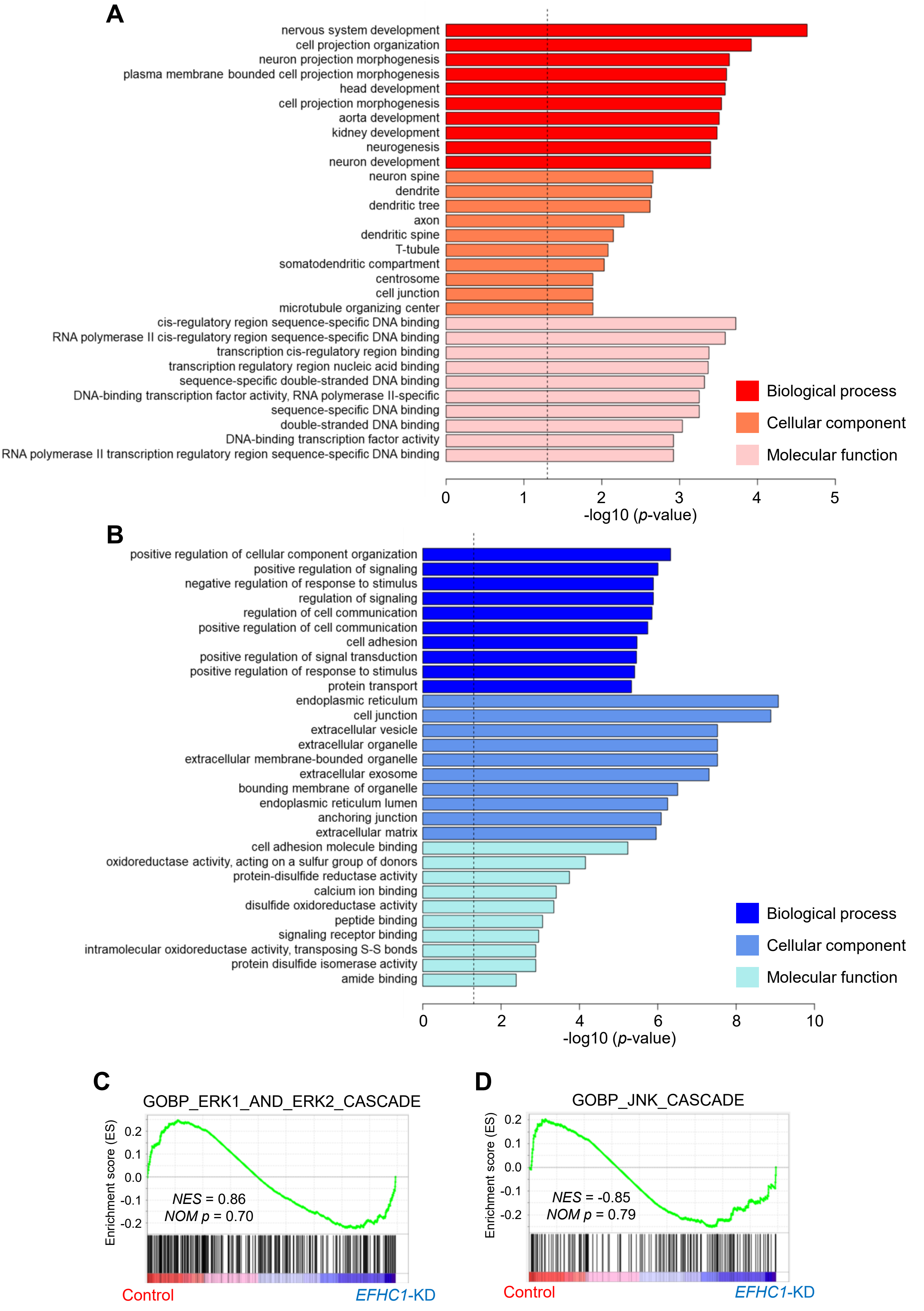

A
nervous system development
cell projection organization
neuron projection morphogenesis
plasma membrane bounded cell projection morphogenesis
head development
cell projection morphogenesis
aorta development
kidney development
neurogenesis
neuron development
neuron spine
dendrite
dendritic tree
axon
dendritic spine
T-tubule
somatodendritic compartment
centrosome
cell junction
microtubule organizing center
cis-regulatory region sequence-specific DNA binding
RNA polymerase II cis-regulatory region sequence-specific DNA binding
transcription cis-regulatory region binding
transcription regulatory region nucleic acid binding
sequence-specific double-stranded DNA binding
DNA-binding transcription factor activity, RNA polymerase II-specific
sequence-specific DNA binding
double-stranded DNA binding
DNA-binding transcription factor activity
RNA polymerase II transcription regulatory region sequence-specific DNA binding
Biological process
Cellular component
Molecular function
0
1
2
3
4
5
-log10 (p-value)
B
positive regulation of cellular component organization
positive regulation of signaling
negative regulation of response to stimulus
regulation of signaling
regulation of cell communication
positive regulation of cell communication
cell adhesion
positive regulation of signal transduction
positive regulation of response to stimulus
protein transport
endoplasmic reticulum
cell junction
extracellular vesicle
extracellular organelle
extracellular membrane-bounded organelle
extracellular exosome
bounding membrane of organelle
endoplasmic reticulum lumen
anchoring junction
extracellular matrix
cell adhesion molecule binding
oxidoreductase activity, acting on a sulfur group of donors
protein-disulfide reductase activity
calcium ion binding
disulfide oxidoreductase activity
peptide binding
signaling receptor binding
intramolecular oxidoreductase activity, transposing S-S bonds
protein disulfide isomerase activity
amide binding
Biological process
Cellular component
Molecular function
0
2
4
6
8
10
-log10 (p-value)
C
GOBP_ERK1_AND_ERK2_CASCADE
Enrichment score (ES)
0.2
0.1
0.0
-0.1
-0.2
NES = 0.86
NOM p = 0.70
Control
EFHC1-KD
D
GOBP_JNK_CASCADE
Enrichment score (ES)
0.2
0.1
0.0
-0.1
-0.2
NES = -0.85
NOM p = 0.79
Control
EFHC1-KD

Fig. S3

***EFHC1* knockdown alters NSC pools, providing more premature neurons, and is enriched in the endoplasmic reticulum.**

(A and B) GO enrichment analysis of DEGs in *EFHC1* knockdown human NSCs. The top 10 enriched GO terms for upregulated (A) and for downregulated (B) DEGs are shown. Gene ontology categories included biological process, cellular component, and molecular function. Dotted lines indicate significance ($p < 0.05$).

(C and D) GSEA of *EFHC1* knockdown AF22 cells for Erk1 and Erk2 cascade (C) and Jnk cascade (D). NES: normalized enrichment score; NOM p: nominal *p*-value.

Fig.S4

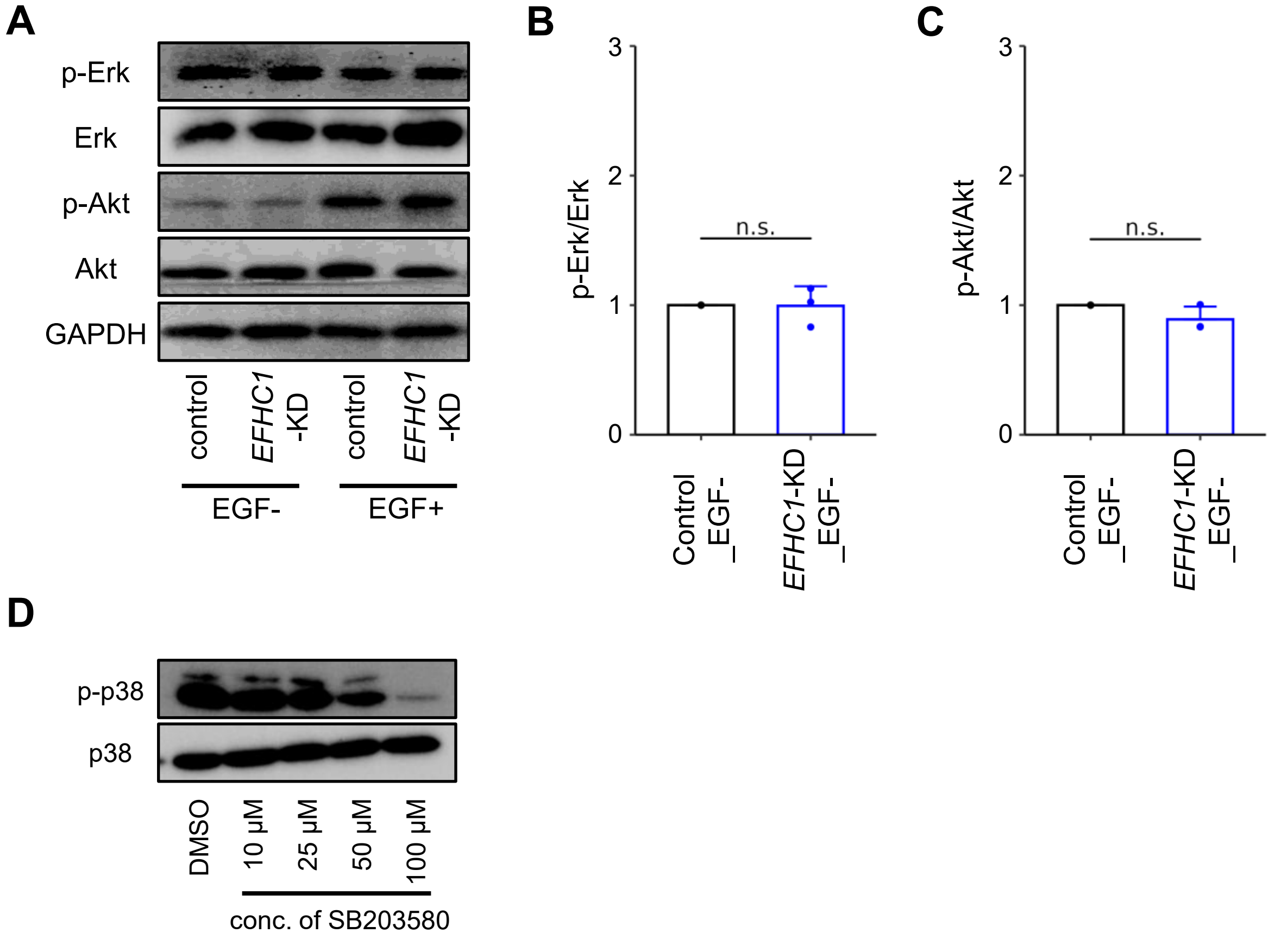
A
p-Erk
Erk
p-Akt
Akt
GAPDH
control
EFHC1 -KD
control
EFHC1 -KD
EGF-
EGF+
B
p-Erk/Erk
n.s.
Control _EGF-
EFHC1-KD _EGF-
C
p-Akt/Akt
n.s.
Control _EGF-
EFHC1-KD _EGF-
D
p-p38
p38
DMSO
10 μM
25 μM
50 μM
100 μM
conc. of SB203580

Fig. S4

***EFHC1* does not affect the activation of Erk or Akt.**

(A) Western blot analysis of whole cell lysates from growth factor-starved control or *EFHC1* knockdown human NSCs treated with EGF 5 min. p-Erk: phospho-Erk, p-Akt: phospho-Akt.

(B and C) Quantification of Erk (B) and Akt (C) phosphorylation levels without EGF in western blot analysis. The values were normalized to control band intensity. n = 3 independent experiments; Student's t test.

(D) Western blot analysis of whole cell lysates from *EFHC1* knockdown human NSCs treated with SB203580 dissolved by DMSO. p-p38: phospho-p38; conc.: concentration.

Fig.S5

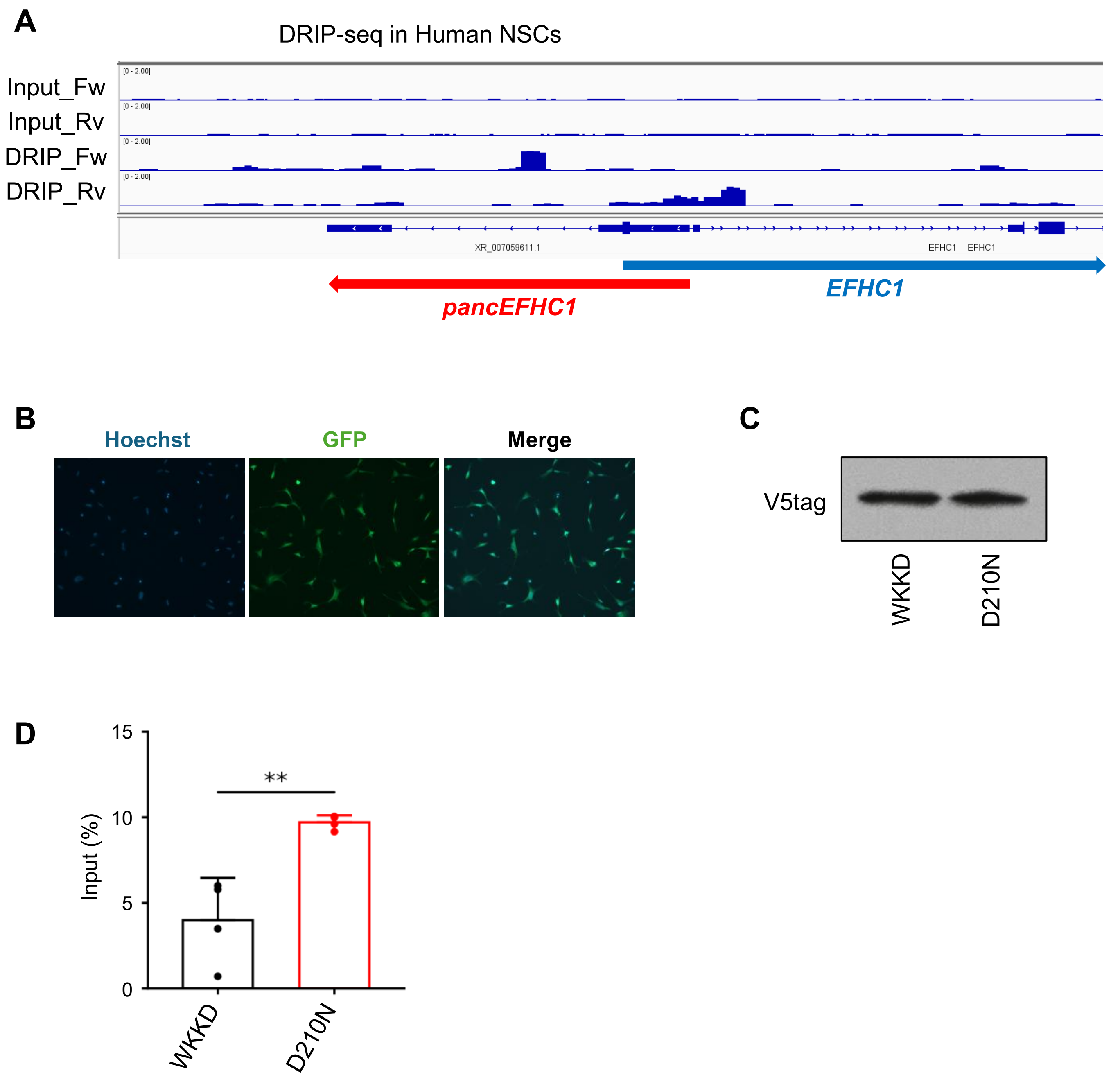
A
DRIP-seq in Human NSCs
Input_Fw
Input_Rv
DRIP_Fw
DRIP_Rv
XR_007059611.1
EFHC1 EFHC1
pancEFHC1
EFHC1
B
Hoechst
GFP
Merge
C
V5tag
WKKD
D210N
D
Input (%)
15
10
5
0
**
WKKD
D210N

Fig. S5

***pancEFHC1* forms an R-loop structure.**

(A) DRIP-seq analysis of forward and reverse strands in human NSCs, showing R-loop peak signals. Blue and red arrows indicate the transcriptional directions of *EFHC1* and *pancEFHC1*, respectively.

(B) Representative images of human NSCs overexpressing RNase H1 mutants and stained for GFP (green), showing nearly 100% infection efficiency. Nuclei were stained with Hoechst (blue).

(C) Western blot analysis of whole cell lysates from human NSCs overexpressing either WKKD or D210N. The expression levels of WKKD and D210N were compared using the V5 tag present in both vectors.

(D) qPCR analysis of purified V5-immunoprecipitated DNA to quantify enrichment at the *EFHC1* locus, shown as percent input). $n = 3$ independent experiments; Student's t test.

mean ± SD; $**P < 0.01$.

Fig.S6

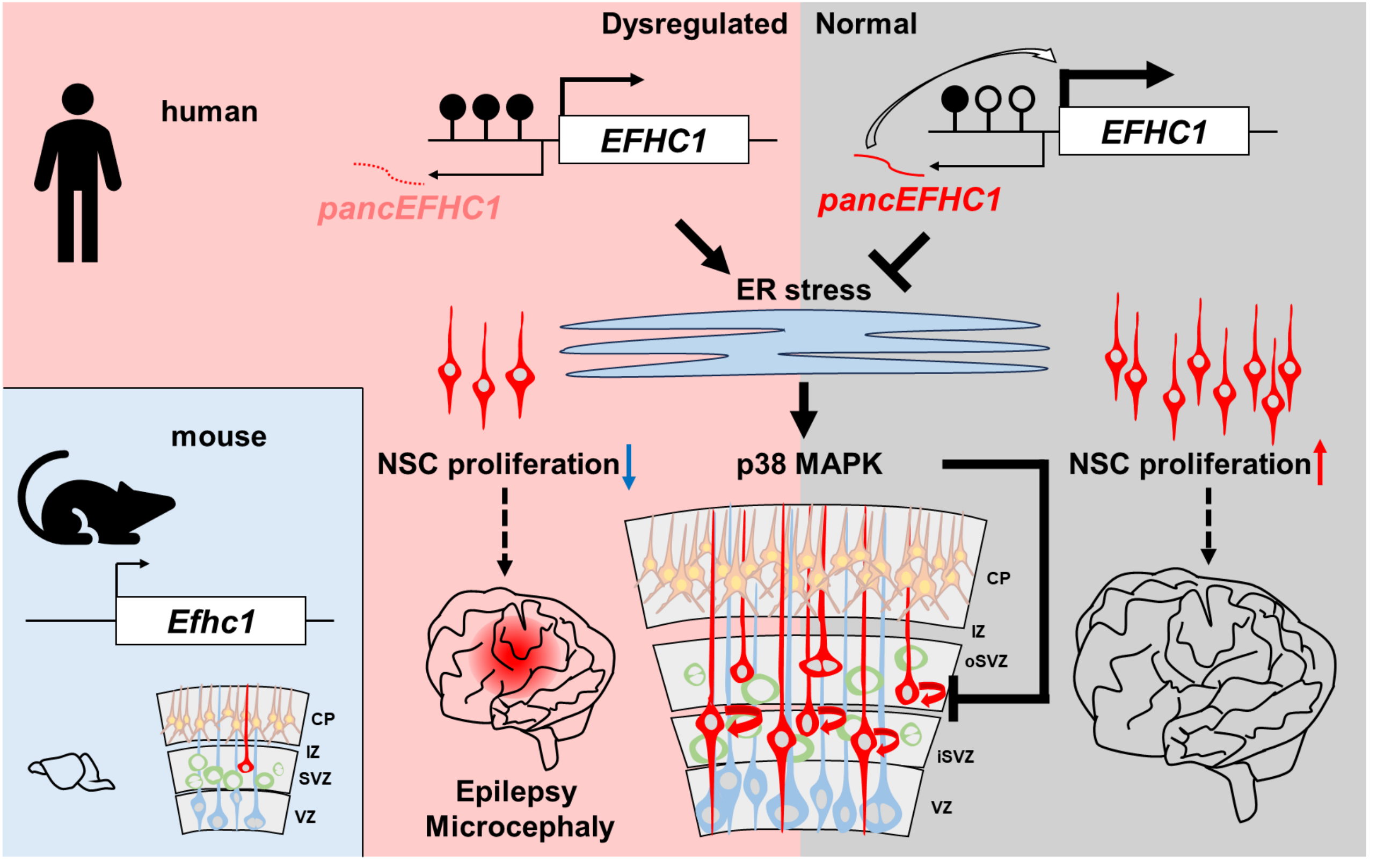

Dysregulated
Normal
human
EFHC1
pancEFHC1
EFHC1
pancEFHC1
ER stress
mouse
NSC proliferation
p38 MAPK
NSC proliferation
Efhc1
CP
IZ
oSVZ
iSVZ
VZ
CP
IZ
SVZ
VZ
Epilepsy
Microcephaly

Fig. S6

Schematic illustration depicting the effects of *pancEFHC1*/*EFHC1* on NSC proliferation and cortical development.
