# Supplementary material for: A human-specific non-coding RNA for EFHC1, an epilepsy-associated gene, regulates neural stem cell proliferation for cortical development
Source: Stem Cell Reports. 2026 Aug 20;21(9):103049. doi: 10.1016/j.stemcr.2026.103049 (PMC13555433; doi:10.1016/j.stemcr.2026.103049)
Supplement: Supplementary file 2 [file mmc3.pdf]

STEM CELL REPORTS
ISSCR
Article
OPEN ACCESS

A human-specific non-coding RNA for *EFHC1*, an epilepsy-associated gene, regulates neural stem cell proliferation for cortical development

Sota Nishida,[1] Boyang An,[1] Hayato Uchida,[1] Shota Zenno,[1] Takumi Nojima,[1] Arisa Makimura,[1] Fumihiro Morishita,[1] Mizuki Honda,[1] and Takuya Imamura[1,2,*]
[1]Program of Biomedical Science, Graduate School of Integrated Sciences for Life, Hiroshima University, Kagamiyama 1-3-1, Higashi-Hiroshima, Hiroshima 739-85216, Japan
[2]Lead contact
*Correspondence: timamura@hiroshima-u.ac.jp
https://doi.org/10.1016/j.stemcr.2026.103049

SUMMARY

Epilepsy is a prevalent brain disorder in humans but rarely occurs naturally in other species, highlighting the potential for human-specific mechanisms in its pathogenesis, and thus, current animal models fail to recapitulate human symptoms. Comparing RNA sequencing (RNA-seq) datasets from human and mouse neural stem cells (NSCs), we identified *EFHC1*, a juvenile myoclonic epilepsy gene, as exhibiting a human-biased expression. *EFHC1* knockdown reduced human NSC proliferation, while its overexpression in mouse embryonic brains increased cortical NSC number. Mechanistically, *EFHC1* prevented endoplasmic reticulum stress, thereby reducing inflammatory activation of p38 MAPK and promoting continuous proliferation of human NSCs. We also identified *pancEFHC1,* a bidirectional promoter-associated non-coding RNA (pancRNA), located at the human *EFHC1* promoter. Knockdown of *pancEFHC1* in human NSCs increased DNA methylation to reduce *EFHC1* expression, with the resulting phenotype rescued by *EFHC1* overexpression. We propose that the evolutionary acquisition of *pancEFHC1* has introduced a complex regulatory mechanism for *EFHC1* expression that allows distinguishing it in humans.

INTRODUCTION

Epilepsy, a brain disease characterized by abnormal and excessive neural activity, leads to recurrent epileptic seizures, affecting as many as 65 million people worldwide (Hagemann et al., 2021; Kanner and Bicchi, 2022). Juvenile myoclonic epilepsy (JME) represents the most common form of idiopathic generalized epilepsy, accounting for 5%–10% of all epilepsy cases. It typically manifests between the ages of 12 and 18 years and involves brief involuntary muscle contractions, tonic-clonic seizures, and, occasionally, absences (Camfield et al., 2013; Delgado-Escueta and Enrile-Bacsal, 1984). JME is a complex genetic disease (Camfield and Camfield, 2009), and several JME-associated genes, including *GABRA1*, *EFHC1*, and *CLCN2*, have been identified (Delgado-Escueta, 2007).

In *EFHC1*, that stands for EF-hand domain containing 1, several missense mutations have been identified in unrelated families with JME probands (Suzuki et al., 2004). The disruption of *EFHC1* has been reported as the most frequent cause of JME (Delgado-Escueta, 2007). EFHC1 is a microtubule-associated 75 kDa protein with three DM10 domains of unknown function and a single EF-hand Ca^{2+}-binding motif (Suzuki et al., 2004). *EFHC1* has been found not only in mammals but also in *Caenorhabditis elegans* (Loucks et al., 2019). For example, the amino acid sequence of *EFHC1/Efhc1* differs by 84.2% between humans and mice. *EFHC1* is pro-apoptotic that enhances Ca^{2+} influx through CaV2.3 voltage-dependent calcium channel in neurons (Suzuki et al., 2004). Interestingly, *EFHC1/Efhc1* is involved not only in the function of terminally differentiated neurons. For instance, *EFHC1* enhances *TRPM2*-mediated susceptibility to H_2O_2-induced cell death, an effect that is reversed by JME mutations (Katano et al., 2012). The motor function of ependymal cell cilia in the brain ventricular wall is decreased in *Efhc1*-knockout mice (Suzuki et al., 2009). In the case of *Efhc1* in rats, it plays a role in neural stem/progenitor/precursor cell division and neuronal migration during cortical development of late pregnancy (de Nijs et al., 2009). This suggests that the various functions of *EFHC1* in different cell types complicate our understanding of its role in epilepsy pathogenesis.

A key to understanding epilepsy is its species-specific nature. While dogs naturally develop epilepsy similar to humans (Ekenstedt et al., 2012), the prevalence is less common in other animals. Various animal models of epilepsy, including genetic models and induced models, have been created (Marshall et al., 2021). However, these models do not fully recapitulate the seizure symptoms, onset time, seizure timing, and frequency observed in human epilepsy. Therefore, epilepsy in humans is likely driven by human-specific onset mechanisms. Comparing human and animal models is expected to ultimately contribute to the elucidation of the pathogenesis of epilepsy, particularly species-dependent features such as the differential proliferative ability of neural stem cells (NSCs) and the resulting variation in neuronal subtypes.

Published by Elsevier Inc. on behalf of International Society for Stem Cell Research.
This is an open access article under the CC BY license (http://creativecommons.org/licenses/by/4.0/).

In this study, we found that *EFHC1/Efhc1* exhibits ~12 times higher expression in human NSCs than in mouse NSCs. The current belief is that the phenotypic diversity is more likely to arise from changes in transcriptional regulation than alterations in protein function (Wray, 2007). Unlike protein-coding regions, gene regulatory regions, such as cell type-specific enhancers, often show more sequence diversity across species. The diversity is likely tolerated because mutations within these regulatory regions typically affect only a limited number of cells, rather than causing systemic effects. Indeed, several genetic regulatory elements influencing the expression of phenotypic differences between species have been identified (Chan et al., 2010; Gompel et al., 2005; Guenther et al., 2014; Jeong et al., 2008; McGregor et al., 2007; Miller et al., 2007). In the context of NSCs, the mechanisms controlling the differential expression as well as functions of EFHC1/Efhc1 proteins have not been elucidated. Their understanding is essential for comprehending the pathogenesis of JME mediated by *EFHC1* in humans.

Here, we show the presence of a bidirectional promoter-associated non-coding RNA (pancRNA) (Imamura et al., 2004) in the human *EFHC1* promoter but not in the mouse counterpart. pancRNA could activate the expression of partner genes through sequence-specific DNA demethylation (Imamura et al., 2004; Tomikawa et al., 2011). We hypothesize that the acquisition of this pancRNA has contributed to the species-specific differences in *EFHC1/Efhc1* expression levels and plays a role in the formation of the enlarged human brain structure that underlies epilepsy onset if pancRNA is deregulated. By identifying and manipulating the *EFHC1*-involving molecular pathway in NSCs, we provide evidence that the acquisition of species-dependent transcriptional regulation, as well as alterations in protein structure, have considerable impacts on NSC proliferation that lead to the alteration of the fetal brain structure.

RESULTS

EFHC1 exhibits the most significant difference in expression among JME-associated genes between humans and mice

In order to narrow down the candidates among epilepsy-related genes from the viewpoint of the human-biased expression, we compared the expression levels of 354 epilepsy-associated genes, obtained from the Harmonizome database, between humans and mice, using published RNA sequencing (RNA-seq) data of NSCs derived from the developing neocortex (Florio et al., 2015). Out of these genes, 218 (approximately 62%) showed higher expression levels in human NSCs (Figure 1A). When we focused on 8 causative genes for JME, we found that *EFHC1* showed the most highly biased expression level toward humans (12-fold compared with mice) (Figures 1B and S1A). We hypothesized that epilepsy can be modeled in mice by tuning the expression patterns of the causative genes close to that of humans. In this scenario, we selected *EFHC1* for the following experiments.

EFHC1 promotes NSC proliferation

To investigate the effect of *EFHC1* on proliferation and apoptosis of human NSCs, we performed knockdown of *EFHC1* in a human induced pluripotent stem cell (iPSC)-derived NSC line, AF22 (Figures 1C and S1B). We immunostained for incorporated 5-ethynyl-2′-deoxyuridine (EdU) to monitor cell cycle progression. We found that *EFHC1* knockdown significantly decreased the ratio of EdU^+ cells compared with the control or scrambled short hairpin RNA (shRNA) groups (Figures 1D and 1E), indicating that *EFHC1* promotes the proliferation of NSCs. We further examined the effect of *EFHC1* on cell survival by immunostaining for active caspase-3. The ratio of active caspase-3^+ cells did not show significant change after *EFHC1* knockdown (Figures 1F and 1G). Taken together, our data indicate that *EFHC1* functions in NSC proliferation but not in NSC survival.

JME-associated genes that are highly expressed in humans play a role in NSC proliferation

In addition to *EFHC1*, we investigated the effects of *GABRG2* and *CACNB4*, which are human-biased genes associated with JME (Figure 1B), by knocking them down in AF22 cells (Figures S1C and S1D). We found that the knockdown of any of these genes significantly decreased the ratio of EdU^+ cells compared with the control group (Figures S1E–S1G), indicating functions similar to *EFHC1* in promoting NSC proliferation. These findings suggest that the multiple human-biased genes associated with JME play a role in the proliferation of NSCs, in addition to their known functions in terminally differentiated neurons.

Human *EFHC1* expression promotes cortical development through increasing NSCs *in vivo*

To explore the function of *EFHC1* in neural development, we induced the expression of *EFHC1* together with GFP in mouse neocortex by *in utero* electroporation on embryonic day 13.5 (E13.5) (Figure 2A). This significantly increased $Sox2^+$ and $Pax6^+$ NSCs in the subventricular zone (SVZ) at E15.5 (Figures 2B–2D and 2F). Similarly, the ratio of $Tbr2^+$ intermediate progenitor cells (IPCs) increased with *EFHC1* expression due to the proliferation of NSCs (Figures 2B–2E). Thus, *EFHC1* promotes the proliferation of NSCs and IPCs *in vivo*. To further investigate the

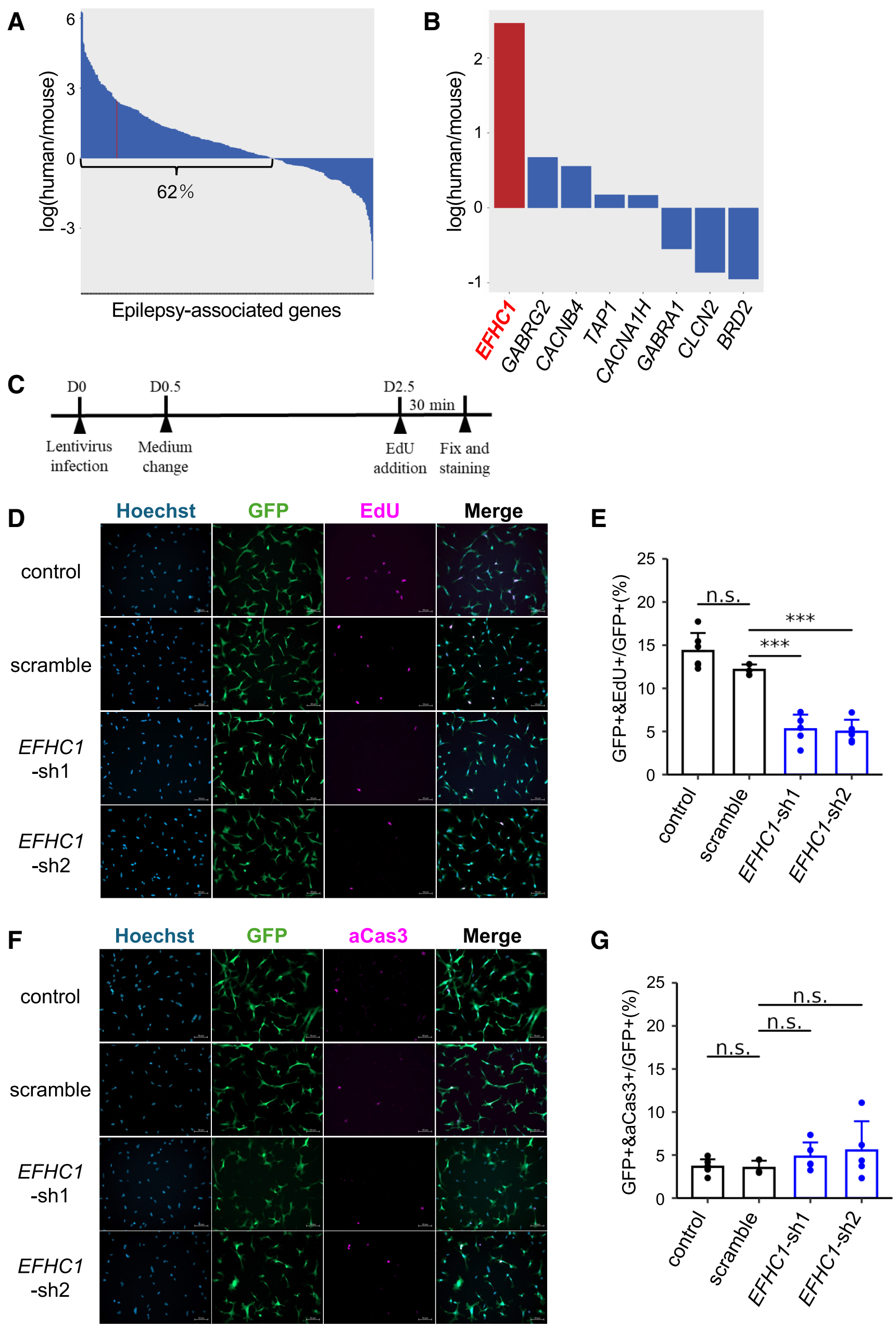

Figure 1. *EFHC1* exhibits the most significant difference in expression among JME-associated genes between humans and mice and affects NSC proliferation

(A and B) Comparison of the expression levels of epilepsy-associated genes (A) and JME-associated genes (B) between human and mouse aRG. The red line indicates *EFHC1*.

(C) An experimental scheme to examine the effect of *EFHC1* knockdown on human NSCs, and NSCs were harvested at the indicated time.

(D) Representative images of human NSCs stained for GFP (green) and EdU (magenta) in control and *EFHC1* knockdown conditions. Nuclei were stained with Hoechst (blue).

(E) Quantification of $EdU^{+}GFP^{+}$ cells to GFP^{+} cells shown in (D). $n = 6$ (control), 5 (*EFHC1*-sh1, *EFHC1*-sh2), and 3 (scramble) independent experiments; data were analyzed using Tukey's test.

(F) Representative images of human NSCs stained for GFP (green) and active caspase-3 (magenta) in control and *EFHC1* knockdown conditions. Nuclei were stained with Hoechst (blue).

(G) Quantification of active caspase-$3^{+}GFP^{+}$ cells to GFP^{+} cells shown in (F). $n = 6$ (control), 5 (*EFHC1*-sh1 and *EFHC1*-sh2), and 3 (scramble) independent experiments; data were analyzed using Tukey's test. Data are presented as mean ± SD; ***, $p < 0.001$; n.s., not significant. Scale bars: 50 μm.

proliferative ability of *EFHC1* in NSCs, we performed phospho-histone H3 (PH3) staining, a marker for M-phase, and observed a significant increase in $PH3^{+}$ cells in the *EFHC1* overexpression group compared with the control at E15.5 (Figures 2C–2G). In addition, to examine the possibility of delamination, we quantified the distribution of GFP^{+} cells across the cortical regions. No significant differences were detected in any region (ventricular zone [VZ], SVZ, intermediate zone [IZ], or cortical plate [CP]) between the control and *EFHC1* overexpression groups (Figures S1H and S1I), indicating that the increase in $Sox2^{+}$ and $Pax6^{+}$ cells in the SVZ reflects enhanced proliferation rather than altered delamination. These results demonstrate that *EFHC1* overexpression enhances NSC proliferation at E15.5. Previous studies have shown that the proliferation of NSCs in SVZ contributes to an increased number of neurons (Heide et al., 2020; Kerimoglu et al., 2021; Liu et al., 2017). To examine the effect of enhanced proliferation caused by *EFHC1* expression on neuronal differentiation, we traced the neuronal populations at a later stage. At

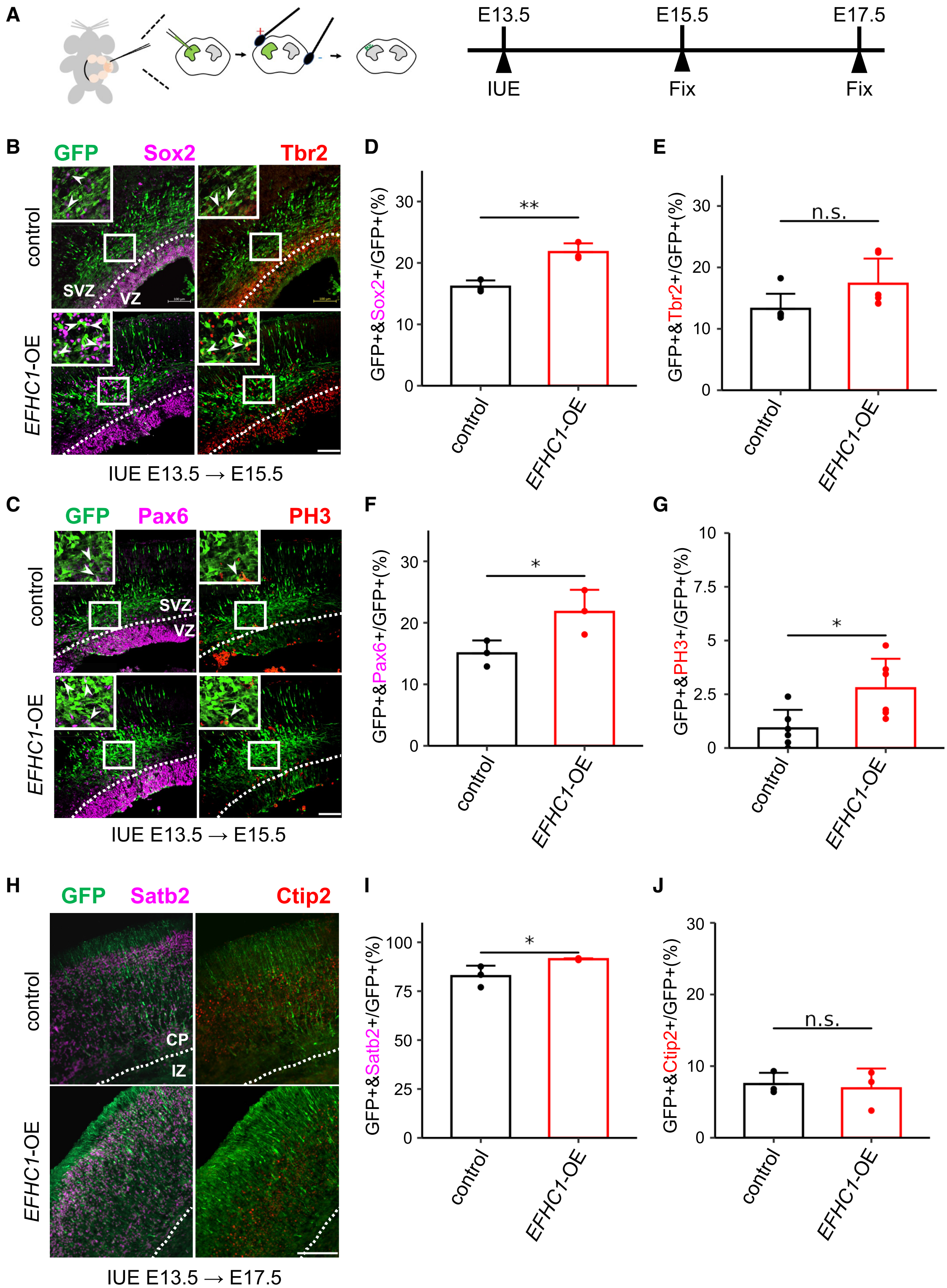

Figure 2. Human *EFHC1* expression promotes cortical development through increasing NSCs *in vivo*

(A) An experimental scheme to examine the effect of *EFHC1* overexpression using *in utero* electroporation.

(B) Representative images of the cortical slices stained for GFP (green), Sox2 (magenta), and Tbr2 (red) in control and *EFHC1*-overexpressing E15.5 developing mice. Nuclei were stained with Hoechst (blue).

(legend continued on next page)

E17.5, immunostaining for Satb2 (a marker for upper-layer neurons) and Ctip2 (a marker for deep-layer neurons) revealed that *EFHC1* expression increased the number of upper-layer neurons, while it did not alter the number of deep-layer neurons. This is likely due, in part, to the fact that many NSCs at E13.5, time of *in utero* electroporation in this study, are fated to become upper-layer neurons (Figures 2H–2J).

Mouse *Efhc1* expression slightly promotes NSC proliferation

As shown in Figure 1B, there is a significant difference in *EFHC1* expression between humans and mice. At the protein level, EFHC1/Efhc1 differs by 84.2% in its amino acid sequence between humans and mice. To investigate whether the structural differences between human EFHC1 and mouse Efhc1 affect the proliferation of NSCs, we conducted an *in vitro* EdU assay using NSCs obtained from E14.5 mouse neocortex after their overexpression. We first confirmed that *EFHC1* and *Efhc1* expression levels were comparable between the respective overexpression vectors (Figure S2A). We found that *Efhc1* overexpression tended to increase the ratio of EdU$^+$ cells compared with the control group (Figures S2B and S2C). However, unlike *EFHC1*, the difference between the control and *Efhc1* groups was not statistically significant (Figures S2B and S2C). This suggests that both increased expression levels and alterations in protein structure have enabled more continuous NSC proliferation in humans. We then examined the overexpression effects *in vivo* and found that while Sox2$^+$ and Pax6$^+$ NSCs tended to increase with mouse *Efhc1* overexpression, it did not show significant effects on proliferation (Figures S2D–S2F and S2H), that contrasted with the data on *EFHC1* overexpression (Figure 2). In addition, *Efhc1* overexpression significantly increased Tbr2$^+$ IPCs compared with the control group, a phenotype not observed with *EFHC1* overexpression (Figures S2D–S2G). This species-dependent divergence suggests that *EFHC1* and *Efhc1* may influence distinct stages of the cortical progenitor hierarchy: *EFHC1* preferentially enhances the expansion or maintenance of Sox2$^+$/Pax6$^+$ NSCs, whereas *Efhc1* more strongly promotes the amplification of Tbr2$^+$ IPCs. These stage-specific effects provide a mechanistic basis for the observed phenotypic differences and further imply that the increased expression of *EFHC1/Efhc1* may promote NSC proliferation in both mice and humans, while the human version of *EFHC1* may have acquired additional regulatory capabilities that contribute to species-specific cortical architecture.

EFHC1 promotes NSC proliferation through the inhibition of p38 MAPK

To explore the signaling pathways regulated by *EFHC1* in NSCs, we performed RNA-seq analysis after *EFHC1* knockdown in AF22 cells. We observed differentially expressed genes (DEGs), of which 322 were upregulated and 484 were downregulated (Figure 3A). Gene Ontology (GO) analysis revealed that the upregulated genes were enriched in biological processes related to nervous system development (GO:0007399) and nerve development (GO:0021675) (Figure S3A), suggesting that *EFHC1* knockdown altered NSC pools to produce more premature neurons. In contrast, the downregulated genes were enriched in biological process related to the regulation of signaling (GO:0023051) and the regulation of cell communication (GO:0010646) (Figure S3B), suggesting that *EFHC1* knockdown affected specific signaling pathways leading to a decrease in cell proliferation. Kyoto Encyclopedia of Genes and Genomes (KEGG) pathway analysis showed that upregulated genes were enriched in the MAPK signaling pathway (Figure 3B). Additionally, gene set enrichment analysis (GSEA) of upregulated genes after *EFHC1* knockdown revealed that those were specifically enriched in p38 MAPK pathway but not in the ERK or JNK pathway (Figures 3C, S3C, and S3D). To validate the possible involvement of p38 MAPK pathway in *EFHC1*-triggered signaling, we conducted western blot analysis. We observed persistent activation of the p38 MAPK signaling pathway in AF22 cells following *EFHC1* knockdown (Figures 3D–3F). Additionally, the increase in p38 phosphorylation was clearly detectable under epidermal growth factor (EGF)-deprived conditions, whereas the difference was not apparent when EGF was present. This is likely

(C) Representative images of the cortical slices stained for GFP (green), Pax6 (magenta), and PH3 (red) in control and *EFHC1*-overexpressing E15.5 developing mice. Nuclei were stained with Hoechst (blue).
(D–G) Quantification of the ratio of Sox2$^+$ (D), Tbr2$^+$ (E), Pax6$^+$ (F), and PH3$^+$ (G) GFP$^+$ cells to GFP$^+$ cells in the SVZ. $n = 3$ (D and F) and 6 (E and G) independent experiments; data were analyzed using Student's t test.
(H) Representative images of the cortical slices stained for GFP (green), Satb2 (magenta), and Ctip2 (red) in control and *EFHC1*-overexpressing E17.5 developing mouse. Nuclei were stained with Hoechst (blue).
(I and J) Quantification of the ratio of Satb2$^+$ (I) and Ctip2$^+$ (J) GFP$^+$ cells to GFP$^+$ cells in the CP. $n = 3$ independent experiments; data were analyzed using Student's t test.
Data are presented as mean ± SD; *, $p < 0.05$; **, $p < 0.01$; n.s., not significant; scale bars: 100 µm.
IUE, *in utero* electroporation; OE, overexpression.

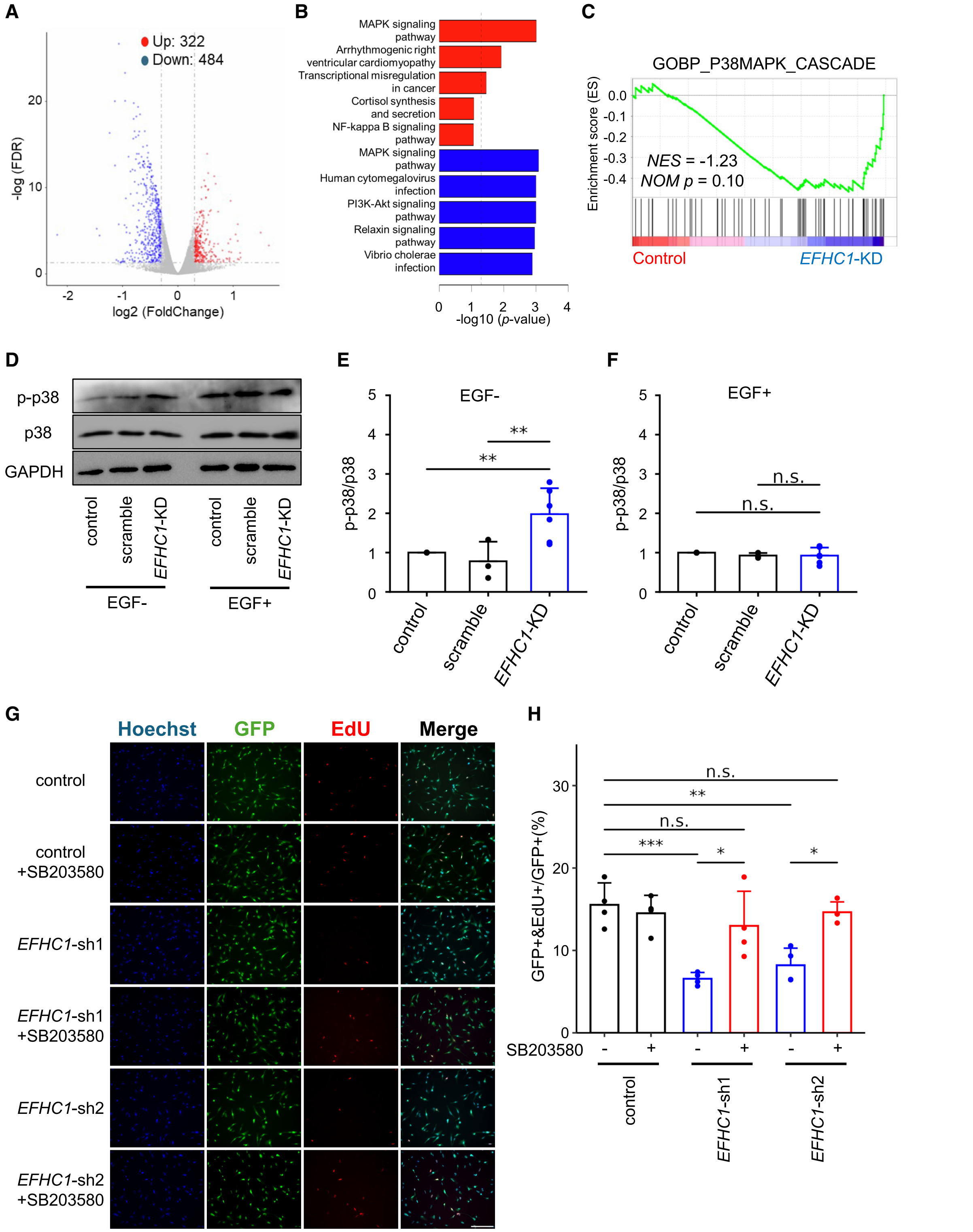

Figure 3. *EFHC1* promotes NSC proliferation through the inhibition of p38 MAPK

(A) Volcano plot showing the merged clusters obtained from RNA-seq analysis of control and *EFHC1*-knockdown human NSCs. Criteria: FDR < 0.05 and $\log_2$ (fold change) > 0.3.

(legend continued on next page)

due to the fact that EGF stimulation elevates basal p38 phosphorylation in control cells, thereby approaching a plateau level at which additional increases induced by *EFHC1* knockdown cannot be readily resolved by western blotting. Consistent with this interpretation, RNA-seq performed under EGF-replete conditions revealed upregulation of p38-associated genes in *EFHC1*-knockdown cells, supporting the notion that *EFHC1* knockdown enhances p38 pathway activity, even when the phosphorylation change is not visually distinguishable in the western blot. In contrast, ERK and AKT signaling pathways, which are known to be involved in G2/M transition of the cell cycle, showed no changes (Figures S4A–S4C). To investigate whether *EFHC1* regulates NSC proliferation through the p38 MAPK signaling pathway, we performed rescue experiments in *EFHC1*-knockdown AF22 cells by the treatment with SB203580, a p38 MAPK inhibitor (Figure S4D). Our results showed that the treatment with SB203580 rescued the decreased ratio of EdU$^+$ cells (Figures 3G and 3H). Collectively, these findings demonstrate that human-type *EFHC1* expression promotes NSC proliferation through inhibition of the p38 MAPK signaling pathway.

EFHC1 knockdown induces ER stress, which reduces NSC proliferation via the activation of p38 MAPK

GO analysis also showed that the downregulated genes were enriched in the cellular component related to the endoplasmic reticulum (ER) (GO:0005783) (Figure S3B), and GSEA indicated that genes involved in protein processing in the ER were affected by *EFHC1* knockdown (Figure 4A). Therefore, we examined the involvement of the ER stress response in *EFHC1*-triggered signaling. We treated *EFHC1* knockdown AF22 NSCs with 4-phenylbutyric acid (4-PBA), an ER stress inhibitor, and the following EdU assay showed that the proliferation defect could be recovered (Figures 4B and 4C). Furthermore, we observed that the phosphorylation level of p38 MAPK, which was activated by *EFHC1* knockdown, was reduced following 4-PBA treatment (Figures 4D–4F). These findings suggest that *EFHC1* prevents ER stress to suppress the inflammatory activation of p38 MAPK, thereby promoting the continuous proliferation of human NSCs.

EFHC1 promotes the proliferation of NSCs through human-specific *pancEFHC1*-mediated gene activation by enhancing demethylation of the *EFHC1* promoter

To investigate the factors contributing to the difference in *EFHC1* expression levels between humans and mice, we visualized RNA-seq data of AF22 NSCs (Brattås et al., 2017) and E15 mouse NSCs (Sakai et al., 2018) by using the Integrative Genomics Viewer (IGV). RNA-seq data revealed that a long non-coding RNA (lncRNA) (Gene: XR_007059611.1) was transcribed in the reverse orientation within the promoter region of *EFHC1* only in humans but not in mice (Figure 5A). Of the 50.4 million and 40.0 million total reads, 41.8 million and 34.5 million uniquely mapped in human and mouse datasets, respectively. We further examined lncRNA expression by using random-hexamer priming during cDNA synthesis in both human and mouse NSCs and detected expression only in human NSCs (Figure 5B). These results indicated that this lncRNA is human specific, and we named it *pancEFHC1*. To investigate the function of *pancEFHC1* on *EFHC1* expression, we conducted quantitative reverse-transcription PCR (RT-qPCR) to assess *EFHC1* expression level, bisulfite PCR to assess DNA methylation levels, and an EdU labeling assay after *pancEFHC1* knockdown in AF22 NSCs. RT-qPCR analysis revealed that *EFHC1* expression was significantly reduced upon *pancEFHC1* knockdown (Figure 5C). Bisulfite PCR further showed that the knockdown of *pancEFHC1* significantly increased DNA methylation in the *EFHC1* promoter region (Figure 5D). Moreover, knockdown of *pancEFHC1* decreased the ratio of EdU$^+$ NSCs, consistent with the data observed following *EFHC1* knockdown (Figures 5E and 5F). We further confirmed that the phenotype triggered by *pancEFHC1* knockdown was rescued by overexpression of *EFHC1* (Figures 5E and 5F). To determine whether *pancEFHC1* regulates downstream processes mediated by *EFHC1*, we treated *pancEFHC1*-knockdown AF22

(B) The top 5 KEGG pathway analysis of upregulated (red) and downregulated (blue) DEGs in *EFHC1*-knockdown human NSCs. Dotted lines indicate significance ($p < 0.05$).

(C) GSEA of *EFHC1*-knockdown AF22 cells for p38 MAPK cascade. NES, normalized enrichment score; NOM p, nominal p value.

(D) Western blot analysis of whole cell lysates from growth factor-starved control or *EFHC1*-knockdown human NSCs treated with EGF for 5 min. p-p38, phospho-p38.

(E and F) Quantification of p38 phosphorylation levels without EGF (E) or with EGF (F) in western blot analysis. The values were normalized to the control band intensity. $n = 6$ (control and *EFHC1*-KD) and 3 (scramble) independent experiments; data were analyzed using Tukey's test.

(G) Representative images of AF22 cells stained for GFP (green) and EdU (red) under control, *EFHC1* knockdown, and *EFHC1* knockdown + SB203580 treatment conditions. Nuclei were stained with Hoechst (blue).

(H) Quantification of the ratio of EdU$^+$GFP$^+$ cells to GFP$^+$ cells shown in (G). $n = 4$ independent experiments; data were analyzed using Tukey's test. Data are presented as mean ± SD; *, $p < 0.05$; **, $p < 0.01$; ***, $p < 0.001$; n.s., not significant. Scale bars: 100 μm.

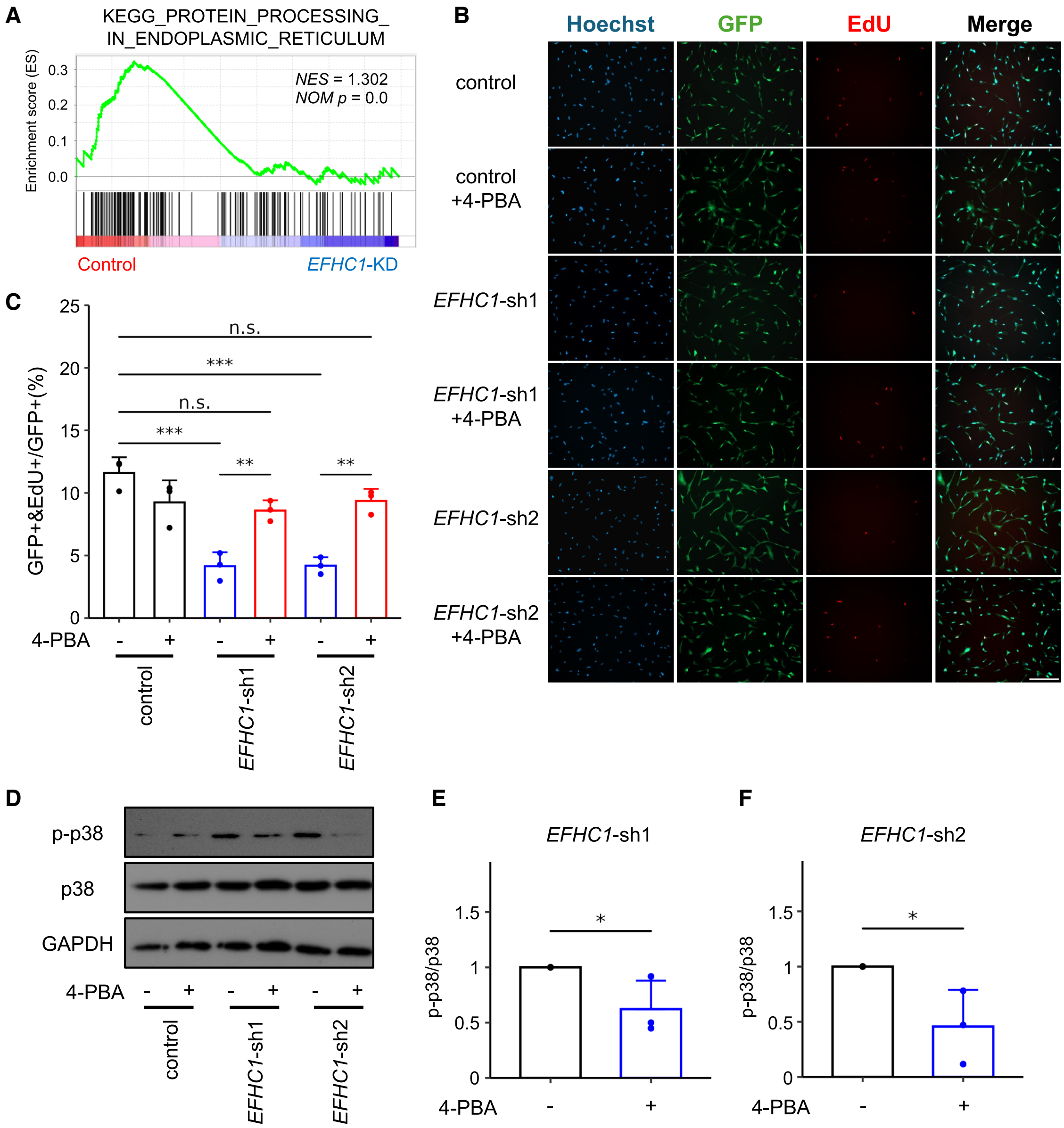

Figure 4. *EFHC1* knockdown induces ER stress, which reduces NSC proliferation via the activation of p38 MAPK

(A) GSEA of *EFHC1*-knockdown AF22 cells for protein processing in the ER. NES, normalized enrichment score; NOM p, nominal *p* value.

(B) Representative images of AF22 cells stained for GFP (green) and EdU (red) under control, *EFHC1* knockdown, and *EFHC1* knockdown + 4-PBA treatment conditions. Nuclei were stained with Hoechst (blue).

(C) Quantification of $EdU^{+}GFP^{+}$ cells to GFP^{+} cells shown in (B). $n = 3$ independent experiments; data were analyzed using Tukey's test.

(D) Western blot analysis of whole cell lysates from growth factor-starved control or *EFHC1*-knockdown human NSCs treated with 4-PBA. p-p38, phospho-p38.

(E and F) Quantification of phosphorylation levels in western blot analysis. The values were normalized to the band intensity of *EFHC1*-knockdown cells untreated with 4-PBA. $n = 3$ independent experiments; data were analyzed using Student's t test. Data are presented as mean ± SD; $^{*}p < 0.05$, $^{**}p < 0.01$, $^{***}p < 0.001$; n.s., not significant. Scale bars: 100 μm.

NSCs with 4-PBA and found that the impaired proliferation was similarly rescued (Figures 5G and 5H). Taken together, our data suggested that a high expression of *EFHC1* promotes the proliferation of human NSCs through human-specific *pancEFHC1*-mediated gene activation by enhancing demethylation of the *EFHC1* promoter.

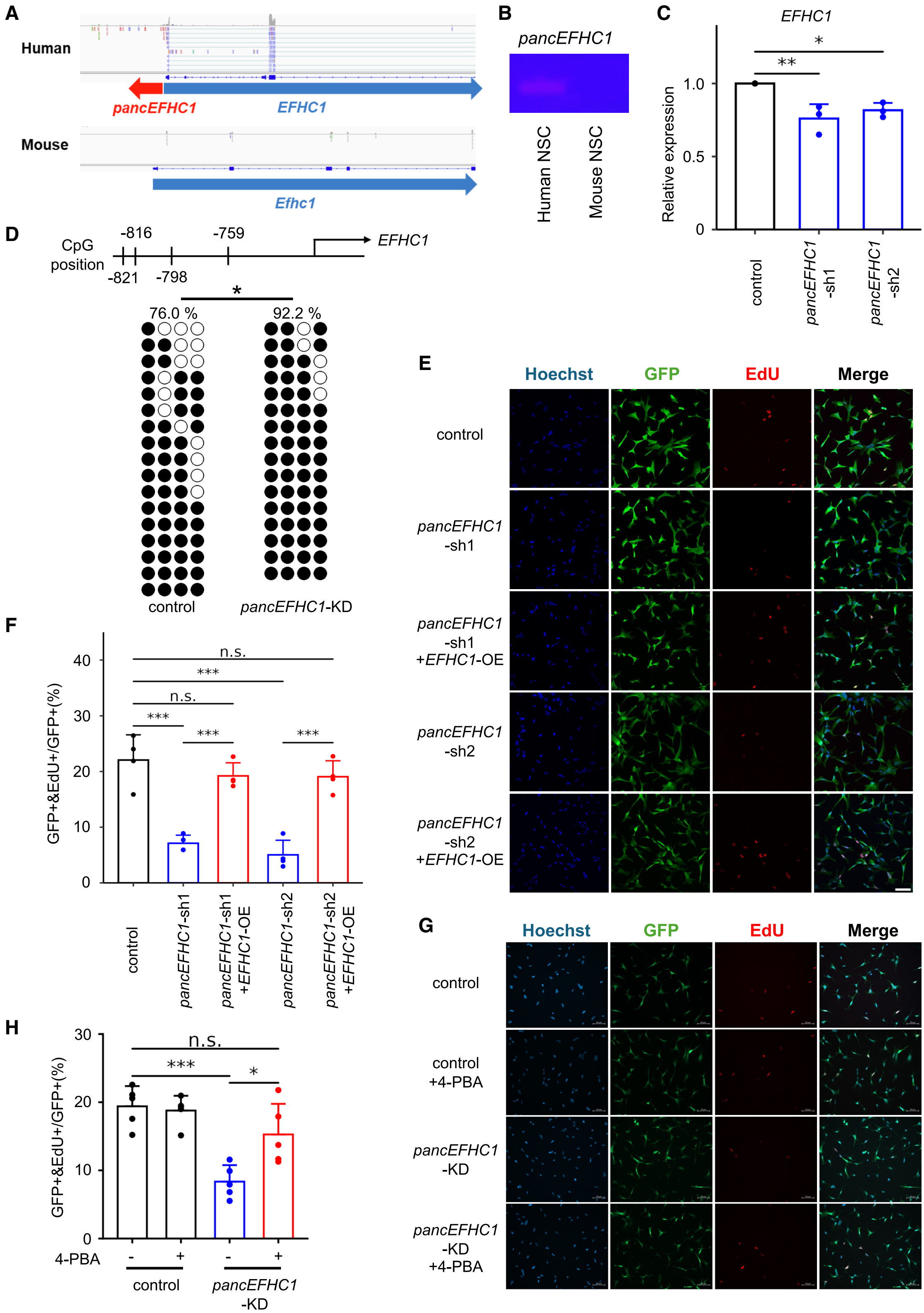

(legend on next page)

pancEFHC1 forms an R-loop structure

A previous study demonstrated that the head-to-head antisense transcript of Vimentin (VIM) promotes the formation of an R-loop structure, thereby enhancing VIM expression (Boque-Sastre et al., 2015). To investigate in greater detail how *EFHC1* is epigenetically regulated, we evaluated the R-loop datasets for human NSCs generated using the DNA-RNA immunoprecipitation sequencing (DRIP-seq) method (Yan et al., 2020). High-intensity R-loop signals were detected at *pancEFHC1*, and notably, the sharp peak was observed at its transcription start site (Figure S5A).

To validate the findings from our genome-wide analysis, we employed RNA-DNA hybrid immunoprecipitation (R-ChIP) assays, a reliable method for detecting R-loop structures (Chen et al., 2019), we induced the overexpression of RNase H1 mutants in AF22 NSCs—D210N (a catalytically inactive, R-loop-binding mutant) and WKKD (a hybrid-binding-deficient mutant used as a negative control)—and confirmed that the transfection efficiency was nearly 100% based on the GFP fluorescence (Figure S5B). Successful expression of the RNase H1 mutants was verified by western blotting, which showed bands at approximately 30–35 kDa (Figure S5C). Using this experimental setup, we performed R-loop pull-down assays at the *pancEFHC1* locus.

Enrichment was quantified by qPCR of purified V5-immunoprecipitated DNA and is expressed as the percent input. We found that the percent input of the D210N mutant was higher than that of the WKKD control (Figure S5D), indicating that R-loop structures were specifically captured at the *pancEFC1* locus. Taken together, these results suggest that *pancEFHC1* is capable of forming R-loop structures.

DISCUSSION

In this study, we found that the evolutionary acquisition of pancRNA has occurred at the *EFHC1* locus that can epigenetically activate the partner gene expression. This mechanism, which selectively upregulates genes in humans, appeared to alter NSC behavior in a way reminiscent of a human-specific trait, such as sustained proliferation leading to increased cell numbers in the developing cortex. Two more JME-involving genes with human-biased expression, *GABRG2* and *CACNB4*, also function in the maintenance of human NSC proliferation, supporting our idea that human-specific features are targeted for JME pathogenesis rather than commonly used mechanisms of NSC proliferation. The mechanisms triggered by pancRNA-induced high expression of *EFHC1* and other genes in human NSCs are discussed below.

Human-biased JME genes as favorable targets for NSC proliferation

Figure S1 shows that all three human-biased genes that are associated with JME and were examined in this study promoted NSC proliferation. These results suggest that epilepsy research should consider human-specific molecular features. In fact, mouse *Efhc1* is known to have a high expression in the epithelial cells of fetal choroid plexus and in postnatal ependymal cells lining the wall of the brain ventricles (Suzuki et al., 2008), and reduced expression of this gene was reported to partially recapitulate JME phenotypes (Suzuki et al., 2009). However, the mutant mice do not develop tonic-clonic seizures, absence seizures, and spike-wave complexes that have been reported in patients with JME. This raises the possibility that additional (environmental, genetic, or species-specific) factors contribute to the appearance of tonic-clonic seizures and absence seizures in humans (Suzuki et al., 2009). Expression of *EFHC1* acquired in the NSCs, in addition to ependymal cells, in humans may have worsened the JME phenotypes. It is worth noting that, while most epilepsy research has focused on neurons, some JME patients exhibit significant structural

Figure 5. *EFHC1* promotes the proliferation of NSCs through human-specific *pancEFHC1*-mediated gene activation by enhancing demethylation of the *EFHC1* promoter

(A) Diagram of the *pancEFHC1*/*EFHC1* locus in humans and mice.

(B) PCR analysis of *pancEFHC1* with random hexamers during cDNA synthesis in both human and mouse NSCs.

(C) Quantification of the mRNA level of *EFHC1* following *pancEFHC1* knockdown. $n = 3$ independent experiments; data were analyzed using Tukey's test.

(D) Top: the location of evaluated CpG sites in the *EFHC1* promoter region. Bottom: the DNA methylation level of individual CpG sites in the *EFHC1* upstream region (−821 to −759) under control and *pancEFHC1* knockdown. Each row of circles represents a single clone. Filled and open circles indicate methylated and unmethylated cytosines at CpG sites, respectively. $n = 17$ (control) and $n = 16$ (*pancEFHC1*-KD) independent experiments; data were analyzed using Mann-Whitney U test; $^*p < 0.05$.

(E) Representative images of human NSCs stained for GFP (green) and EdU (red) under control, *pancEFHC1* knockdown, and *pancEFHC1* knockdown and *EFHC1* overexpression conditions. Nuclei were stained with Hoechst (blue).

(F) Quantification of EdU^+GFP^+ cells to GFP^+ cells shown in (E). $n = 4$ independent experiments; data were analyzed using Tukey's test.

(G) Representative images of AF22 cells stained for GFP (green) and EdU (red) under control, *pancEFHC1* knockdown, and *pancEFHC1* knockdown + 4-PBA treatment conditions. Nuclei were stained with Hoechst (blue).

(H) Quantification of EdU^+GFP^+ cells to GFP^+ cells shown in (G). $n = 5$ independent experiments; data were analyzed using Tukey's test. Data are presented as mean ± SD; $^*p < 0.05$, $^{**}p < 0.01$, $^{***}p < 0.001$; n.s., not significant. Scale bars are 100 μm (E) and 50 μm (G).

abnormalities of cortical gray matter (Woermann et al., 1999), and a subset of JME patients with *EFHC1* mutations suffer from microcephaly (Berger et al., 2012). Our data, combined with these findings, suggest that *EFHC1* can function in early NSCs and/or surrounding cells in a human-specific manner. In line with this idea, abnormal NSC behaviors may contribute to JME phenotypes in humans; however, it may be difficult to model these features in other mammalian species. Nevertheless, there is evidence of *Efhc1* functionalization even in rodent NSCs. In the late pregnancy rat brain, where NSC expansion declines as precursor differentiation into glial cells begins, *Efhc1* knockout results in cortical precursors failing to exit the cell cycle, as well as defects in the radial glia (RG) scaffold organization and the locomotion of postmitotic neurons (de Nijs et al., 2009). We propose that in addition to late-stage NSCs and their surrounding cells, early-stage NSCs have emerged as key targets of JME genes, contributing to human cortical expansion via increased human-specific expression.

Quantitative and qualitative regulation of *EFHC1* for driving human-specific phenomena

Previous studies have reported that not only human-specific genes but also widely conserved genes contribute to the acquisition of the human-specific higher brain. For example, *TMEM25* is expressed at much higher levels in human NSCs than in mouse NSCs and promotes basal RG proliferation, which facilitates neuron production and supports cortical development (An et al., 2023). Additionally, *TKTL1* exhibits a human-specific function caused by only a single amino acid substitution compared with Neanderthals, increasing the abundance of basal RG and leading to the generation of more neocortical neurons during neocortical neurogenesis (Pinson et al., 2022). In the case of human *EFHC1,* it is expressed at much higher levels and has evolved additional capabilities to alter cortical structures compared with its mouse counterpart through alterations in its protein structure (Figures S1 and S2). A previous study showed that EFHC1 directly interacts with microtubules via this N-terminal region, thereby regulating cell division in HEK293 cells (de Nijs et al., 2009). In actuality, 7 amino acid substitutions are observed within the N-terminal region, which may influence how EFHC1/Efhc1 regulates NSC proliferation. These findings represent the first demonstration that *EFHC1* has evolved under synergistic quantitative and qualitative regulation, making it a key contributor to the acquisition of the human-specific higher brain.

Intracellular mechanisms of human-specific proliferation of NSCs triggered by *EFHC1*

As shown in Figures 3 and 4, we found that *EFHC1* prevented ER stress to reduce inflammatory activation of p38 MAPK for continuous proliferation of human NSCs. The ER is the primary organelle for protein synthesis, folding, and Ca^{2+} storage. Changes in ER luminal Ca^{2+} homeostasis, regulated by Ca^{2+} channels and other mechanisms, can influence the quality and efficiency of protein folding, potentially leading to ER stress (Krebs et al., 2015; Michalak et al., 2002). In *EFHC1*, in addition to the presence of the EF-hand Ca^{2+}-binding motif, several functions related to cellular Ca^{2+} homeostasis have been reported. Specifically, *EFHC1* enhances Ca^{2+} influx into the cell through CaV2.3 voltage-dependent calcium channel in neurons (Suzuki et al., 2004) and potentiates the activity of TRPM2, a Ca^{2+}-permeable cation channel, when co-expressed in HEK293 cells (Katano et al., 2012). Therefore, it is possible that EFHC1 interacts with or regulates channels that maintain ER Ca^{2+} homeostasis, such as the inositol 1,4,5-trisphosphate receptor and the ryanodine receptor, thereby suppressing ER stress. Previous studies have shown that ER stress induces the activation of p38 MAPK in mouse midbrain dopaminergic progenitor cells (Li et al., 2017) and human gingival cells (Kim et al., 2010). In neurospheres derived from adult hippocampal NSCs, p38α, a subunit of p38 MAPK, regulates neuronal differentiation, and inhibition of p38α maintains the NSC stemness (Yoshioka et al., 2015). p38 MAPK functions as a negative regulator of NSC proliferation during early brain development (Sato et al., 2008). Additionally, activated p38 MAPK is known to produce proinflammatory cytokines (Bachstetter and Van Eldik, 2010), and excessive production of these cytokines in NSCs can lead to brain developmental disorders. For example, Zika virus infection reduces NSC proliferation and impairs neurogenesis, leading to microcephaly in fetuses, with inflammatory cytokines playing a critical role in the pathogenesis (Li et al., 2016; Wen et al., 2017). Therefore, it is highly probable that *EFHC1* promotes the maintenance of stemness in NSCs by inhibiting p38 MAPK through the regulation of ER Ca^{2+} homeostasis. Compared with mice, humans maintain their stemness for a longer period, which leads to the acquisition of a huge number of neurons and brain expansion (Florio and Huttner, 2014). We propose that the species difference in *EFHC1* discussed above has conferred an advantage in maintaining NSC stemness by inhibiting the ER stress-p38 MAPK axis.

The acquisition of human-biased expression of *EFHC1* by human-specific non-coding RNA

Our findings indicated that the evolutionary acquisition of *pancEFHC1* was key to achieving the human-biased expression of *EFHC1*, which is essential for the maintenance of stemness in NSCs. Figure 5 shows that *pancEFHC1* is exclusively present in the human genome and activates *EFHC1* expression by enhancing demethylation of the *EFHC1* promoter, thereby leading to increased cell proliferation. This gene-activating property is consistent with the broader functional characteristics of pancRNAs. We previously

demonstrated that pancRNAs specifically upregulate the expression of their partner mRNAs. For example, *pancNusap1* activates *Nusap1* through histone acetylation (Yamamoto et al., 2016), and *pancIl17d* enhances demethylation of the *Il17d* promoter by recruiting a methylcytosine hydroxylase TET3 and a base excision repair enzyme PARP (Hamazaki et al., 2015). Other groups also have validated the gene activating functions of rodent pancRNAs that we discovered in human counterparts in *VIM* and *SPHK1* loci (Imamura et al., 2004; Tomikawa et al., 2011), suggesting that pancRNA functions together with transcription factors to upregulate the partner gene expression. In addition to these epigenetic mechanisms, R-loop formation appears to represent another layer of pancRNA-mediated gene activation. The head-to-head antisense transcript of *VIM* has been shown to promote the recruitment of transcriptional activators through R-loop formation (Boque-Sastre et al., 2015). By analogy, our data suggest that *pancEFHC1* may similarly form an R-loop at the *EFHC1* promoter, thereby facilitating the binding of transcriptional activators and contributing to *EFHC1* upregulation. The evolutionary context of *pancEFHC1* further highlights the unique regulatory potential of pancRNAs. Transcription factors are often well conserved among species due to the preservation of the corresponding DNA sequence (Carroll, 2005), whereas pancRNAs are much more diverged depending on species (Uesaka et al., 2017), although some lncRNAs also exhibit high sequence and expression pattern conservation, as represented by Tuna (a conserved lncRNA essential for maintaining pluripotency and neural differentiation in mouse embryonic stem cells), which regulates the transcription of thousands of genes (Lin et al., 2014). Nevertheless, promoter sequences often exhibit high variability, reflecting their tolerance to genomic changes. This variability can lead to the gain or loss of pancRNAs to upregulate partner genes. Indeed, the diversity of pancRNA expression profiles is greater than that of mRNA expression profiles (Uesaka et al., 2017). Taking into account that pancRNA repertoires are highly species specific compared with protein-coding genes, our data suggest that the acquisition of human-specific pancRNAs may have led to a sudden increase in the expression levels of certain conserved genes during the evolutionary expansion of the human cortex.

Conclusion

Our study reveals that the pancRNA-mediated upregulation of *EFHC1* plays a critical role in the human-specific expansion of NSCs and cortical development. By suppressing ER stress and p38 MAPK activation, *EFHC1* maintains NSC stemness, facilitating prolonged proliferation and increased neuron production in humans (Figure S6). The acquisition of human-specific pancRNAs, such as *pancEFHC1*, and minute alterations in protein structure have enabled the functional enhancement of genes like *EFHC1*, contributing to the expansion of the human cortex. These findings provide valuable insights into the molecular mechanisms underlying human brain evolution and highlight potential pathways involved in the etiology of neurological disorders such as JME.

RESOURCE AVAILABILITY

Lead contact

Requests for further information, resources, reagents, and data for reanalysis should be directed to the lead contact, Takuya Imamura (timamura@hiroshima-u.ac.jp).

Materials availability

Materials are available upon reasonable request to the lead contact.

Data and code availability

The raw sequencing data have been deposited in Gene Expression Omnibus under accession number GEO: GSE286261.

ACKNOWLEDGMENTS

We are grateful to Dr. Tatsuya Ueki for insightful discussions. We thank members of the Imamura laboratory for their suggestions during this work. This work was supported, in part, by the Natural Science Center for Basic Research and Development (NBARD-00122). This study was supported by grants from JSPS KAKENHI (grant numbers 22H02531, 19H03138, and 24K21918 [to T.I.]), NOVARTIS Foundation (Japan) for the Promotion of Science, Uehara Memorial Foundation, Daiichi Sankyo Foundation of Life Science, Mitsubishi Foundation, and Kobayashi Foundation.

AUTHOR CONTRIBUTIONS

S.N. and T.I. designed the research; T.I. supervised the project and secured the funding; S.N., B.A., H.U., S.Z., T.N., M.H., and T.I. performed experiments, interpreted results, and wrote the manuscript. All members commented on and have edited and revised the manuscript.

DECLARATION OF INTERESTS

The authors declare no competing interests.

STAR★METHODS

Detailed methods are provided in the online version of this paper and include the following:

- KEY RESOURCES TABLE
- EXPERIMENTAL MODEL AND STUDY PARTICIPANTS DETAILS
  - Mice
  - Human NSC culture
  - Mouse NSC culture
- METHOD DETAILS
  - Constructs

- ○ Lentivirus production
- ○ Reverse transcription and qRT-PCR analysis
- ○ *In utero* electroporation
- ○ Immunocytochemistry and Immunohistochemistry
- ○ EdU assays
- ○ Bisulfite sequencing
- ○ RNA sequencing and data analysis
- ○ Western blotting
- ○ R-ChIP and DNA recovery
- QUANTIFICATION AND STATISTICAL ANALYSIS

SUPPLEMENTAL INFORMATION

Supplemental information can be found online at https://doi.org/10.1016/j.stemcr.2026.103049.

Received: February 20, 2025
Revised: July 17, 2026
Accepted: July 18, 2026
Published: August 20, 2026

REFERENCES

An, B., Ando, A., Akuta, H., Morishita, F., and Imamura, T. (2023). Human-biased TMEM25 expression promotes expansion of neural progenitor cells to alter cortical structure in the developing brain. FEBS Lett. *597*, 2611–2625. https://doi.org/10.1002/1873-3468.14756.

Bachstetter, A.D., and Van Eldik, L.J. (2010). The p38 MAP kinase family as regulators of proinflammatory cytokine production in degenerative diseases of the CNS. Aging Dis. *1*, 199–211.

Berger, I., Dor, T., Halvardson, J., Edvardson, S., Shaag, A., Feuk, L., and Elpeleg, O. (2012). Intractable epilepsy of infancy due to homozygous mutation in the EFHC1 gene: Intractable Epilepsy EFHC1 Mutation. Epilepsia *53*, 1436–1440. https://doi.org/10.1111/j.1528-1167.2012.03536.x.

Boque-Sastre, R., Soler, M., Oliveira-Mateos, C., Portela, A., Moutinho, C., Sayols, S., Villanueva, A., Esteller, M., and Guil, S. (2015). Head-to-head antisense transcription and R-loop formation promotes transcriptional activation. Proc. Natl. Acad. Sci. USA *112*, 5785–5790. https://doi.org/10.1073/pnas.1421197112.

Brattås, P.L., Jönsson, M.E., Fasching, L., Nelander Wahlestedt, J., Shahsavani, M., Falk, R., Falk, A., Jern, P., Parmar, M., and Jakobsson, J. (2017). TRIM28 controls a gene regulatory network based on endogenous retroviruses in human neural progenitor cells. Cell Rep. *18*, 1–11. https://doi.org/10.1016/j.celrep.2016.12.010.

Camfield, C.S., and Camfield, P.R. (2009). Juvenile myoclonic epilepsy 25 years after seizure onset: a population-based study. Neurology *73*, 1041–1045. https://doi.org/10.1212/WNL.0b013e3181b9c86f.

Camfield, C.S., Striano, P., and Camfield, P.R. (2013). Epidemiology of juvenile myoclonic epilepsy. Epilepsy Behav. *28*, S15–S17. https://doi.org/10.1016/j.yebeh.2012.06.024.

Carroll, S.B. (2005). Evolution at two levels: on genes and form. PLoS Biol. *3*, e245. https://doi.org/10.1371/journal.pbio.0030245.

Chan, Y.F., Marks, M.E., Jones, F.C., Villarreal, G., Jr., Shapiro, M.D., Brady, S.D., Southwick, A.M., Absher, D.M., Grimwood, J., Schmutz, J., et al. (2010). Adaptive evolution of pelvic reduction in sticklebacks by recurrent deletion of a Pitx1 enhancer. Science *327*, 302–305. https://doi.org/10.1126/science.1182213.

Chen, J.-Y., Zhang, X., Fu, X.-D., and Chen, L. (2019). R-ChIP for genome-wide mapping of R-loops by using catalytically inactive RNASEH1. Nat. Protoc. *14*, 1661–1685. https://doi.org/10.1038/s41596-019-0154-6.

Delgado-Escueta, A.V. (2007). Advances in genetics of juvenile myoclonic epilepsies. Epilepsy Curr. *7*, 61–67. https://doi.org/10.1111/j.1535-7511.2007.00171.x.

Delgado-Escueta, A.V., and Enrile-Bacsal, F. (1984). Juvenile myoclonic epilepsy of Janz. Neurology *34*, 285–294. https://doi.org/10.1212/wnl.34.3.285.

Dobin, A., Davis, C.A., Schlesinger, F., Drenkow, J., Zaleski, C., Jha, S., Batut, P., Chaisson, M., and Gingeras, T.R. (2013). STAR: ultrafast universal RNA-seq aligner. Bioinformatics *29*, 15–21. https://doi.org/10.1093/bioinformatics/bts635.

Ekenstedt, K.J., Patterson, E.E., and Mickelson, J.R. (2012). Canine epilepsy genetics. Mamm. Genome *23*, 28–39. https://doi.org/10.1007/s00335-011-9362-2.

Falk, A., Koch, P., Kesavan, J., Takashima, Y., Ladewig, J., Alexander, M., Wiskow, O., Tailor, J., Trotter, M., Pollard, S., et al. (2012). Capture of neuroepithelial-like stem cells from pluripotent stem cells provides a versatile system for in vitro production of human neurons. PLoS One *7*, e29597.

Florio, M., and Huttner, W.B. (2014). Neural progenitors, neurogenesis and the evolution of the neocortex. Development *141*, 2182–2194. https://doi.org/10.1242/dev.090571.

Florio, M., Albert, M., Taverna, E., Namba, T., Brandl, H., Lewitus, E., Haffner, C., Sykes, A., Wong, F.K., Peters, J., et al. (2015). Human-specific gene ARHGAP11B promotes basal progenitor amplification and neocortex expansion. Science *347*, 1465–1470. https://doi.org/10.1126/science.aaa1975.

Gompel, N., Prud'homme, B., Wittkopp, P.J., Kassner, V.A., and Carroll, S.B. (2005). Chance caught on the wing: cis-regulatory evolution and the origin of pigment patterns in Drosophila. Nature *433*, 481–487. https://doi.org/10.1038/nature03235.

Guenther, C.A., Tasic, B., Luo, L., Bedell, M.A., and Kingsley, D.M. (2014). A molecular basis for classic blond hair color in Europeans. Nat. Genet. *46*, 748–752. https://doi.org/10.1038/ng.2991.

Hagemann, A., Wilting, J., Samimizad, B., Mormann, F., and Priesemann, V. (2021). Assessing criticality in pre-seizure single-neuron activity of human epileptic cortex. PLoS Comput. Biol. *17*, e1008773. https://doi.org/10.1371/journal.pcbi.1008773.

Hamazaki, N., Uesaka, M., Nakashima, K., Agata, K., and Imamura, T. (2015). Gene activation-associated long noncoding RNAs function in mouse preimplantation development. Development *142*, 910–920. https://doi.org/10.1242/dev.116996.

Heide, M., Haffner, C., Murayama, A., Kurotaki, Y., Shinohara, H., Okano, H., Sasaki, E., and Huttner, W.B. (2020). Human-specific ARHGAP11B increases size and folding of primate neocortex in the fetal marmoset. Science *369*, 546–550. https://doi.org/10.1126/science.abb2401.

Imamura, T., Yamamoto, S., Ohgane, J., Hattori, N., Tanaka, S., and Shiota, K. (2004). Non-coding RNA directed DNA demethylation

of Sphk1 CpG island. Biochem. Biophys. Res. Commun. *322*, 593–600. https://doi.org/10.1016/j.bbrc.2004.07.159.

Imamura, T., Kerjean, A., Heams, T., Kupiec, J.-J., Thenevin, C., and Pàldi, A. (2005). Dynamic CpG and non-CpG methylation of the Peg1/Mest gene in the mouse oocyte and preimplantation embryo. J. Biol. Chem. *280*, 20171–20175. https://doi.org/10.1074/jbc.M501749200.

Jeong, S., Rebeiz, M., Andolfatto, P., Werner, T., True, J., and Carroll, S.B. (2008). The evolution of gene regulation underlies a morphological difference between two Drosophila sister species. Cell *132*, 783–793. https://doi.org/10.1016/j.cell.2008.01.014.

Kanner, A.M., and Bicchi, M.M. (2022). Antiseizure medications for adults with epilepsy: A review: A review. JAMA *327*, 1269–1281. https://doi.org/10.1001/jama.2022.3880.

Katano, M., Numata, T., Aguan, K., Hara, Y., Kiyonaka, S., Yamamoto, S., Miki, T., Sawamura, S., Suzuki, T., Yamakawa, K., and Mori, Y. (2012). The juvenile myoclonic epilepsy-related protein EFHC1 interacts with the redox-sensitive TRPM2 channel linked to cell death. Cell Calcium *51*, 179–185. https://doi.org/10.1016/j.ceca.2011.12.011.

Kerimoglu, C., Pham, L., Tonchev, A.B., Sakib, M.S., Xie, Y., Sokpor, G., Ulmke, P.A., Kaurani, L., Abbas, E., Nguyen, H., et al. (2021). H3 acetylation selectively promotes basal progenitor proliferation and neocortex expansion. Sci. Adv. *7*, eabc6792. https://doi.org/10.1126/sciadv.abc6792.

Kim, D.-S., Kim, J.-H., Lee, G.-H., Kim, H.-T., Lim, J.M., Chae, S.-W., Chae, H.-J., and Kim, H.-R. (2010). p38 Mitogen-activated protein kinase is involved in endoplasmic reticulum stress-induced cell death and autophagy in human gingival fibroblasts. Biol. Pharm. Bull. *33*, 545–549. https://doi.org/10.1248/bpb.33.545.

Krebs, J., Agellon, L.B., and Michalak, M. (2015). Ca(2+) homeostasis and endoplasmic reticulum (ER) stress: An integrated view of calcium signaling. Biochem. Biophys. Res. Commun. *460*, 114–121. https://doi.org/10.1016/j.bbrc.2015.02.004.

Langmead, B., and Salzberg, S.L. (2012). Fast gapped-read alignment with Bowtie 2. Nat. Methods *9*, 357–359. https://doi.org/10.1038/nmeth.1923.

Li, C., Xu, D., Ye, Q., Hong, S., Jiang, Y., Liu, X., Zhang, N., Shi, L., Qin, C.-F., and Xu, Z. (2016). Zika virus disrupts neural progenitor development and leads to microcephaly in mice. Cell Stem Cell *19*, 120–126. https://doi.org/10.1016/j.stem.2016.04.017.

Li, H., Handsaker, B., Wysoker, A., Fennell, T., Ruan, J., Homer, N., Marth, G., Abecasis, G., and Durbin, R.; 1000 Genome Project Data Processing Subgroup (2009). The Sequence Alignment/Map format and SAMtools. Bioinformatics *25*, 2078–2079. https://doi.org/10.1093/bioinformatics/btp352.

Li, W., Zhu, J., Dou, J., She, H., Tao, K., Xu, H., Yang, Q., and Mao, Z. (2017). Phosphorylation of LAMP2A by p38 MAPK couples ER stress to chaperone-mediated autophagy. Nat. Commun. *8*, 1763. https://doi.org/10.1038/s41467-017-01609-x.

Lin, N., Chang, K.-Y., Li, Z., Gates, K., Rana, Z.A., Dang, J., Zhang, D., Han, T., Yang, C.-S., Cunningham, T.J., et al. (2014). An evolutionarily conserved long noncoding RNA TUNA controls pluripotency and neural lineage commitment. Mol. Cell *53*, 1005–1019. https://doi.org/10.1016/j.molcel.2014.01.021.

Liu, J., Liu, W., Yang, L., Wu, Q., Zhang, H., Fang, A., Li, L., Xu, X., Sun, L., Zhang, J., et al. (2017). The primate-specific gene TMEM14B marks outer radial Glia cells and promotes cortical expansion and folding. Cell Stem Cell *21*, 635–649.e8. https://doi.org/10.1016/j.stem.2017.08.013.

Lois, C., Hong, E.J., Pease, S., Brown, E.J., and Baltimore, D. (2002). Germline transmission and tissue-specific expression of transgenes delivered by lentiviral vectors. Science *295*, 868–872. https://doi.org/10.1126/science.1067081.

Loucks, C.M., Park, K., Walker, D.S., McEwan, A.H., Timbers, T.A., Ardiel, E.L., Grundy, L.J., Li, C., Johnson, J.-L., Kennedy, J., et al. (2019). EFHC1, implicated in juvenile myoclonic epilepsy, functions at the cilium and synapse to modulate dopamine signaling. eLife *8*, e37271. https://doi.org/10.7554/eLife.37271.

Marshall, G.F., Gonzalez-Sulser, A., and Abbott, C.M. (2021). Modelling epilepsy in the mouse: challenges and solutions. Dis. Model. Mech. *14*, dmm047449. https://doi.org/10.1242/dmm.047449.

McGregor, A.P., Orgogozo, V., Delon, I., Zanet, J., Srinivasan, D.G., Payre, F., and Stern, D.L. (2007). Morphological evolution through multiple cis-regulatory mutations at a single gene. Nature *448*, 587–590. https://doi.org/10.1038/nature05988.

Michalak, M., Robert Parker, J.M., and Opas, M. (2002). Ca2+ signaling and calcium binding chaperones of the endoplasmic reticulum. Cell Calcium *32*, 269–278. https://doi.org/10.1016/s0143416002001884.

Miller, C.T., Beleza, S., Pollen, A.A., Schluter, D., Kittles, R.A., Shriver, M.D., and Kingsley, D.M. (2007). cis-Regulatory changes in Kit ligand expression and parallel evolution of pigmentation in sticklebacks and humans. Cell *131*, 1179–1189. https://doi.org/10.1016/j.cell.2007.10.055.

Nakashima, H., Tsujimura, K., Irie, K., Ishizu, M., Pan, M., Kameda, T., and Nakashima, K. (2018). Canonical TGF-β signaling negatively regulates neuronal morphogenesis through TGIF/Smad complex-mediated CRMP2 suppression. J. Neurosci. *38*, 4791–4810. https://doi.org/10.1523/JNEUROSCI.2423-17.2018.

de Nijs, L., Léon, C., Nguyen, L., Loturco, J.J., Delgado-Escueta, A.V., Grisar, T., and Lakaye, B. (2009). EFHC1 interacts with microtubules to regulate cell division and cortical development. Nat. Neurosci. *12*, 1266–1274. https://doi.org/10.1038/nn.2390.

Pinson, A., Xing, L., Namba, T., Kalebic, N., Peters, J., Oegema, C.E., Traikov, S., Reppe, K., Riesenberg, S., Maricic, T., et al. (2022). Human TKTL1 implies greater neurogenesis in frontal neocortex of modern humans than Neanderthals. Science *377*, eabl6422. https://doi.org/10.1126/science.abl6422.

Quinlan, A.R., and Hall, I.M. (2010). BEDTools: a flexible suite of utilities for comparing genomic features. Bioinformatics *26*, 841–842. https://doi.org/10.1093/bioinformatics/btq033.

Ramírez, F., Dündar, F., Diehl, S., Grüning, B.A., and Manke, T. (2014). deepTools: a flexible platform for exploring deep-sequencing data. Nucleic Acids Res. *42*, W187–W191. https://doi.org/10.1093/nar/gku365.

Sakai, A., Matsuda, T., Doi, H., Nagaishi, Y., Kato, K., and Nakashima, K. (2018). Ectopic neurogenesis induced by prenatal antiepileptic drug exposure augments seizure susceptibility in adult mice.

Proc. Natl. Acad. Sci. USA *115*, 4270–4275. https://doi.org/10.1073/pnas.1716479115.

Sato, K., Hamanoue, M., and Takamatsu, K. (2008). Inhibitors of p38 mitogen-activated protein kinase enhance proliferation of mouse neural stem cells. J. Neurosci. Res. *86*, 2179–2189. https://doi.org/10.1002/jnr.21668.

Suzuki, T., Delgado-Escueta, A.V., Aguan, K., Alonso, M.E., Shi, J., Hara, Y., Nishida, M., Numata, T., Medina, M.T., Takeuchi, T., et al. (2004). Mutations in EFHC1 cause juvenile myoclonic epilepsy. Nat. Genet. *36*, 842–849. https://doi.org/10.1038/ng1393.

Suzuki, T., Inoue, I., Yamagata, T., Morita, N., Furuichi, T., and Yamakawa, K. (2008). Sequential expression of Efhc1/myoclonin1 in choroid plexus and ependymal cell cilia. Biochem. Biophys. Res. Commun. *367*, 226–233. https://doi.org/10.1016/j.bbrc.2007.12.126.

Suzuki, T., Miyamoto, H., Nakahari, T., Inoue, I., Suemoto, T., Jiang, B., Hirota, Y., Itohara, S., Saido, T.C., Tsumoto, T., et al. (2009). Efhc1 deficiency causes spontaneous myoclonus and increased seizure susceptibility. Hum. Mol. Genet. *18*, 1099–1109. https://doi.org/10.1093/hmg/ddp006.

Tomikawa, J., Shimokawa, H., Uesaka, M., Yamamoto, N., Mori, Y., Tsukamura, H., Maeda, K.-I., and Imamura, T. (2011). Single-stranded noncoding RNAs mediate local epigenetic alterations at gene promoters in rat cell lines. J. Biol. Chem. *286*, 34788–34799. https://doi.org/10.1074/jbc.M111.275750.

Tsujimura, K., Irie, K., Nakashima, H., Egashira, Y., Fukao, Y., Fujiwara, M., Itoh, M., Uesaka, M., Imamura, T., Nakahata, Y., et al. (2015). MiR-199a links MeCP2 with mTOR signaling and its dysregulation leads to Rett syndrome phenotypes. Cell Rep. *12*, 1887–1901. https://doi.org/10.1016/j.celrep.2015.08.028.

Uesaka, M., Agata, K., Oishi, T., Nakashima, K., and Imamura, T. (2017). Evolutionary acquisition of promoter-associated non-coding RNA (pancRNA) repertoires diversifies species-dependent gene activation mechanisms in mammals. BMC Genom. *18*, 285. https://doi.org/10.1186/s12864-017-3662-1.

Wen, Z., Song, H., and Ming, G.-L. (2017). How does Zika virus cause microcephaly? Genes Dev. *31*, 849–861. https://doi.org/10.1101/gad.298216.117.

Woermann, F.G., Free, S.L., Koepp, M.J., Sisodiya, S.M., and Duncan, J.S. (1999). Abnormal cerebral structure in juvenile myoclonic epilepsy demonstrated with voxel-based analysis of MRI. Brain *122*, 2101–2108. https://doi.org/10.1093/brain/122.11.2101.

Wray, G.A. (2007). The evolutionary significance of cis-regulatory mutations. Nat. Rev. Genet. *8*, 206–216. https://doi.org/10.1038/nrg2063.

Yamamoto, N., Agata, K., Nakashima, K., and Imamura, T. (2016). Bidirectional promoters link cAMP signaling with irreversible differentiation through promoter-associated non-coding RNA (pancRNA) expression in PC12 cells. Nucleic Acids Res. *44*, 5105–5122. https://doi.org/10.1093/nar/gkw113.

Yan, P., Liu, Z., Song, M., Wu, Z., Xu, W., Li, K., Ji, Q., Wang, S., Liu, X., Yan, K., et al. (2020). Genome-wide R-loop landscapes during cell differentiation and reprogramming. Cell Rep. *32*, 107870. https://doi.org/10.1016/j.celrep.2020.107870.

Yoshioka, K., Namiki, K., Sudo, T., and Kasuya, Y. (2015). P38α controls self-renewal and fate decision of neurosphere-forming cells in adult hippocampus. FEBS Open Bio *5*, 437–444. https://doi.org/10.1016/j.fob.2015.05.001.

Zhang, Y., Liu, T., Meyer, C.A., Eeckhoute, J., Johnson, D.S., Bernstein, B.E., Nusbaum, C., Myers, R.M., Brown, M., Li, W., and Liu, X.S. (2008). Model-based analysis of ChIP-Seq (MACS). Genome Biol. *9*, R137. https://doi.org/10.1186/gb-2008-9-9-r137.

Zhou, Z., Hong, E.J., Cohen, S., Zhao, W.-N., Ho, H.-Y.H., Schmidt, L., Chen, W.G., Lin, Y., Savner, E., Griffith, E.C., et al. (2006). Brain-specific phosphorylation of MeCP2 regulates activity-dependent Bdnf transcription, dendritic growth, and spine maturation. Neuron *52*, 255–269. https://doi.org/10.1016/j.neuron.2006.09.037.

STAR★METHODS

KEY RESOURCES TABLE

REAGENT or RESOURCE	SOURCE	IDENTIFIER
**Antibodies**		
Chick anti GFP	Aves Labs	Cat#: GFP-1020; RRID:AB_2307313
Rabbit anti-active caspase3	R&D Systems	Cat#: AF835; RRID:AB_2243952
Rabbit anti-Pax6	Biolegend	Cat#: 901302; RRID:AB_256003
Mouse anti-phospho-histone H3	Cell Signaling	Cat#: 9706; RRID: AB_331748
Goat anti-Sox2	R&D Systems	Cat#: AF2018; RRID: AB_355110
Rabbit anti-Tbr2	abcam	Cat#: ab23345; RRID: AB_778267
Mouse anti- SATB2	abcam	Cat#: ab51502; RRID: AB_882455
Rat anti-Ctip2	abcam	Cat#: ab18465; RRID:AB_2064130
Rabbit anti-phospho-p38 MAPK	Cell Signaling	Cat#: 4511; RRID:AB_2139682
Rabbit anti-p38 MAPK	Cell Signaling	Cat#: 8690; RRID: AB_10999090
Rabbit anti-phospho-p44/42 MAPK (Erk1/2)	Cell Signaling	Cat#: 9101; RRID:AB_331646
Rabbit anti-p44/42 MAPK (Erk1/2)	Cell Signaling	Cat#: 9102; RRID:AB_330744
Rabbit anti-phospho-Akt	Cell Signaling	Cat#: 4060; RRID:AB_2315049
Rabbit anti-Akt	Cell Signaling	Cat#: 9272; RRID:AB_329827
Rabbit anti-GAPDH	Cell Signaling	Cat#: 5174; RRID:AB_10622025
Rabbit anti-V5-tag	Cell Signaling	Cat#: 13202; RRID:AB_2687461
Mouse anti FLAG	Sigma-Aldrich	Cat#: F1804; RRID:AB_262044
**Bacterial and virus strains**		
E. coli DH5a	This study	N/A
**Chemicals, peptides, and recombinant proteins**		
bFGF	PeproTech	Cat#: 10018B
EGF	PeproTech	Cat#: AF10015
SB203580	abcam	Cat#: ab12062
4-Phenylbutanoic acid	Angene	Cat#: AG0023DD
**Critical commercial assays**		
ReverTra Ace® qPCR RT Master Mix with gDNA Remover	Toyobo	Cat#: FSQ-301
THUNDERBIRD Next SYBR qPCR Mix	Toyobo	Cat#: QPX-201
EZ DNA Methylation-Gold kit	Zymo Research	Cat#: D5005
AmpliTaq GoldTM 360 DNA Polymerase	Thermo Scientific	Cat#: 4398813
NEBNext Ultra II Directional RNA library Prep Kit for Illumina	New England and Biolabs	Cat#: E7760

(*Continued on next page*)

Continued

REAGENT or RESOURCE	SOURCE	IDENTIFIER
**Deposited data**		
Raw sequencing data	This study	GEO: GSE286261
**Experimental models: Cell lines**		
HEK293T	ATCC	Cat#: CRL-3216
Mouse NSCs	This study	N/A
Human NSCs: AF22 cell line	Gift from Austin Smith	N/A
**Experimental models: Organisms/strains**		
Mouse: ICR	Japan SLC	N/A
**Oligonucleotides**		
Primers for qRT-PCR, see Table S1	This study	N/A
Primers for shRNA, see Table S1	This study	N/A
**Recombinant DNA**		
pLLX	Lois et al. (2002); Zhou et al. (2006)	N/A
pLEMPRA	Lois et al. (2002); Zhou et al. (2006)	N/A
**Software and algorithms**		
ImageJ	NIH	https://imagej.net/ij/
ZEN microscope software	ZEISS	https://www.zeiss.com/corporate/en/home.html

EXPERIMENTAL MODEL AND STUDY PARTICIPANTS DETAILS

Mice

All aspects of animal care and treatment were performed according to the guidelines of the Experimental Animal Care Committee of Hiroshima University (Hiroshima, Japan). Timed pregnant ICR mice (Japan SLC) were used in this study.

Human NSC culture

The human NSC line AF22 was established and maintained in monolayer culture as previously described(Falk et al., 2012). AF22 was cultured in poly-L-ornithine (Sigma, P3655) and laminin (RSD, 3400-010-02) coated dish, using hN2 medium (DMEM/F12 (Nacalai Tesque, 846095), 100 μg/mL apo-transferrin (Nacalai Tesque, 3440155), 2.5 μg/mL insulin (Fujifilm, 97–6474), 16 μg/mL putrescine dihydrochloride (Sigma, P5780), 30 μM sodium selenite (Sigma, S5261), 20 μM progesterone (Sigma, P8783) supplemented with 1 μL/mL B27 (Invitrogen, 17504001), 10 ng/mL bFGF (PeproTech, 10018B), 10 ng/mL EGF (PeproTech, AF10015) and penicillin/streptomycin/fungizone (PSF; Nacalai Tesque, 2892-54). The cells were passaged at a ratio of 1:2 every third or fourth day using TrypLE Select (Gibco, 12563029).

For the rescue experiments, the p38 MAPK inhibitor SB203580 (10 μM; Abcam, ab120162) or endoplasmic reticulum (ER) stress inhibitor 4-Phenylbutanoic acid (10 μM; Angene, AG0023DD) was added 1 day after infection and remained present for 1 day. To detect the downstream signaling, the cells were cultured in hN2 medium without insulin or growth factors for 2h. Following starvation, the cells were treated with 10 ng/mL EGF for 5 min or 10 μM 4-Phenylbutanoic acid for 1h.

Mouse NSC culture

Mouse NSCs were isolated from embryonic day 14.5 (E14.5) mouse embryo (male and female) telencephalons according to a previously described protocol.(Nakashima et al., 2018) In brief, the embryo telencephalons were triturated in Hanks' balanced salt solution (HBSS; Sigma, H2387) by gentle pipetting. After centrifugation, dissociated cells were cultured in poly-L-ornithine and fibronectin (Sigma) coated dish, using mN2 medium (DMEM/F12, 100 μg/mL apo-transferrin,

25 μg/mL insulin, 16 μg/mL putrescine dihydrochloride, 30 μM sodium selenite, 20 μM progesterone) supplemented with 10 ng/mL bFGF and PSF.

All cells were maintained at 37°C under a humidified atmosphere containing 5% CO_2.

METHOD DETAILS

Constructs

Lentivirus vectors used to express short hairpin RNAs (shRNAs; pLLX) and FLAG-tagged MeCP2 (pLEMPRA)(Lois et al., 2002; Zhou et al., 2006) were provided by Dr. Z. Zhou (University of Pennsylvania School of Medicine, Philadelphia, PA, USA) and Dr. M. E. Greenberg (Harvard Medical School, Boston, MA, USA). An FLAG-tagged human and mouse *EFHC1* expression lentivirus vector was constructed by replacing MeCP2 with *EFHC1* at the EcoRI and AscI sites of pLEMPRA-MeCP2. shRNAs against *EFHC1* and *pancEFHC1* were designed to target human genes.

To enable R-loop capture by R-ChIP, human RNASEH1 variants were expressed with a nuclear localization signal and a C-terminal V5 tag. The catalytically inactive D210N mutant was used as an R-loop–binding variant, whereas the hybrid-binding–deficient WKKD mutant (W43A/K59A/K60A/D210N) served as a negative control.

Lentivirus production

Lentivirus were generated as described previously.(Tsujimura et al., 2015) Briefly, lentivirus particles were produced by co-transfecting HEK293T cells with the lentivirus constructs pCMV-VSV-G-RSV-Rev and pCAG-HIVgp using polyethyleneimine (PEI; Polysciences, 23966-1). The culture supernatants were collected 48h after transfection, and virus was introduced into human or mouse NSCs by adding these supernatants to the culture medium.

Reverse transcription and qRT-PCR analysis

Total RNAs were extracted with Sepasol-RNA I Super G (Nacalai Tesque, 9379-97) and subsequently reverse transcribed with the ReverTra Ace qPCR RT Master Mix with gDNA Remover (Toyobo, FSQ-301), according to the manufacturer's instructions. To detect the presence of pancRNA, Random Hexamers Primer (BMS, BRSM7020100) was added before denaturation.

qRT-PCR was performed using a THUNDERBIRD Next SYBR qPCR Mix (Toyobo, QPX-201) on a CronoSTAR 96 Real-Time PCR System (Clontech, 640231). The gene expression levels were normalized to that of GAPDH and calculated relative to the control group. PCR primers used in this study are listed in Table S1.

In utero electroporation

In utero electroporation was performed as described previously.(Nakashima et al., 2018) Briefly, electroporation was performed on E13.5 mouse embryos (male and female). Plasmid DNA (1.0 μg/mL) with 0.1% Fast Green was injected into the lateral ventricle of the embryonic brain from outside the uterus using a calibrated glass micropipette. For electroporation, 50 ms electric pulses of 40 V were delivered five times at intervals of 950 ms using a Super Electroporator NEPA21 Type II (Nepa Gene). Following the procedure, the uterus was returned to the abdominal cavity, and the wound was surgically sutured. Embryonic brains were fixed by perfusion with 4% paraformaldehyde at E15.5 or E17.5. Sequentially, fixed brains were dehydrated in 10% and 20% sucrose in PBS at 4°C.

Immunocytochemistry and Immunohistochemistry

Cells were fixed with 4% paraformaldehyde and washed 3 times with PBS. Cells or brain sections were then permeabilized and blocked with blocking buffer (0.1% Triton X-100 and 3% FBS in PBS) for 20 min at room temperature (RT) and incubated with primary antibodies diluted in blocking buffer at RT for 2 h or at 4°C overnight. The following primary antibodies were used in this study: chick anti-GFP (1: 500; Aves Labs, GFP1020); rabbit anti-active caspase3 (1: 500; R&D, AF835); rabbit anti-Pax6 (1: 500; Biolegend, 901302); mouse anti-phospho-histone H3 (1: 500; CST, 9706); goat anti-Sox2 (1: 500; R&D, AF2018); rabbit anti-Tbr2 (1: 500; Abcam, ab23345); mouse anti-Satb2 (1: 500; Abcam, ab51502); rat anti-Ctip2 (1: 500; Abcam, ab18465). After being washed 3 times with PBS, the cells were incubated at RT for 2h with corresponding secondary antibodies: CF488A donkey anti-chicken IgY (IgG) (H + L), highly cross-adsorbed (1: 500; Biotium, Fremont, CA, USA); CF555 donkey anti-mouse IgG (H + L), highly cross-adsorbed (1: 500; Biotium); CF555 donkey anti-rabbit IgG (H + L), highly cross-adsorbed (1: 500; Biotium); CF568 donkey anti-rat IgG (H + L), highly cross-adsorbed (1: 500; Biotium); CF647 donkey anti-goat IgG (H + L), highly cross-adsorbed (1: 500; Biotium); CF647 donkey anti-rabbit IgG (H + L), highly cross-adsorbed (1: 500; Biotium); or CF647 donkey anti-mouse IgG (H + L), highly

cross-adsorbed (1: 500; Biotium). Hoechst 33342 (1: 500; Nacalai Tesque, 19172-51) was used for nuclear staining. After a final washing with PBS, they were mounted on glass slides with Immu-Mount (Thermo Scientific, 9990412). Fluorescence images were acquired using an FV1000-D (Olympus), an FV3000 (Olympus) or a ZEISS Axio Observer (ZEISS) with a 20× objective lens.

EdU assays

Cells were cultured with 10 μM EdU in Click-iT EdU Imaging Kits (Thermo Scientific, C10338) for 30 min, fixed with 4% paraformaldehyde, and stained with Click-iT reaction buffer at RT for 40 min in the dark. After washing with PBS, primary and secondary staining were performed in the dark.

Bisulfite sequencing

To determine the DNA methylation profile of the *EFHC1* promoter region, genomic DNA from human NSCs was subjected to the bisulfite reaction using EZ DNA Methylation-Gold kit (ZYR, D5005) according to the manufacturer's instructions. Each bisulfite-treated genome was amplified using AmpliTaq Gold™ 360 DNA Polymerase (Thermo Scientific, 4398813) and specific primers. To avoid PCR bias, we subcloned three PCR bands following previously described,(Imamura et al., 2005) and performed bisulfite sequencing on at least 16 resulting subclones from each sample. Sequences were analyzed using the methylation analysis tool QUantification tool for Methylation Analysis (QUMA: http://quma.cdb.riken.jp). The statistical significance of differences between the two groups of the entire CpG sites was evaluated using Mann-Whitney *U* test.

RNA sequencing and data analysis

Total RNAs were extracted using Sepasol-RNA I Super G and the NucleoSpin RNA kit (Takara, 740955). mRNAs were then further isolated with NEBNext Poly(A) mRNA Magnetic Isolation Module (New England Biolabs, E7490) according to the manufacturer's standard procedure. Purified mRNAs were supplied for library construction using the NEBNext Ultra II Directional RNA library Prep Kit for Illumina (New England and Biolabs, E7760) according to the manufacturer's protocols. cDNA libraries were size-selected with AMPure XP beads (Beckman Coulter, A63881). cDNA library qualities were checked using Agilent 2100 Bioanalyser (Agilent Technologies). RNA sequencing was performed with 150-bp paired-end sequencing using a DNBSEQ-G400 (BGI). Adaptor and incomplete sequences were removed by Trim Galore. Short-read sequences were mapped on the human reference genome hg38 by STAR.(Dobin et al., 2013) Mapped reads were counted by BEDTools.(Quinlan and Hall, 2010) To compare gene expression changes, TPM values were utilized.

Public strand-specific DRIP-seq datasets for hNSCs (SRR11185387 and SRR11185388) and the corresponding input control (SRR12462635) were obtained from the SRA.(Yan et al., 2020) Adapter and quality trimming was performed using Trim Galore v0.6.6. Trimmed reads were aligned to the GRCh38/hg38 reference genome using bowtie2 v2.5.4,(Langmead and Salzberg, 2012) and alignments were coordinate sorted using samtools v1.22.1.(Li et al., 2009) PCR duplicates were removed using MarkDuplicates v3.4.0. For strand-specific analyses, strand assignment was based on Read 1 (library-specific strandedness), and strand-specific BAM files (forward/Watson and reverse/Crick) were generated accordingly. Strand-specific coverage tracks were generated using deepTools bamCoverage v3.5.1 with CPM normalization (–normalizeUsing CPM) and 50-bp binning (–binSize 50) and exported as bigWig files.(Ramírez et al., 2014) Strand-specific broad peaks were called independently for each replicate against the strand-matched input control using MACS2 v2.2.6 with a q-value cutoff of 0.01 and broad-peak mode (–broad -q 0.01 –broad-cutoff 0.01).(Zhang et al., 2008) Model building was disabled (–nomodel), and reads were extended to a fixed fragment length of 250 bp (–extsize 250) to match the fragment size described in the reference protocol (sonicated to 250-bp fragments). For downstream analyses, forward and reverse peak sets were concatenated and merged using bedtools merge (bedtools v2.29.2), and a combined peak set across SRR11185387 and SRR11185388 was generated by concatenation followed by merging.(Quinlan and Hall, 2010) Overlaps with genomic annotations were quantified using bedtools intersect.(Quinlan and Hall, 2010).

Western blotting

Proteins were extracted using RIPA buffer (Thermo Scientific, 89900) with 1% protease inhibitor (Nacalai Tesque, 25955-11), then separated by SDS-PAGE and transferred to PVDF membranes (Millipore, IPVH15150). Following transfer, the membranes were cut based on molecular mass and blocked with 5% Bovine serum albumin (Sigma, A9418) at RT for 1 h. The membranes were incubated with primary antibodies at 4°C overnight. The following primary antibodies were used in this study: phosphor-p38 MAPK (1 : 1000; CST, 4511); p38 MAPK (1 : 1000; CST, 8690); phosphor-Erk (1 : 1000; CST, 9101); Erk (1 : 1000; CST, 9102); phospho-Akt (1 : 2000; CST, 4060); Akt (1 : 1000; CST, 9272); GAPDH (1 :

1000; CST, 5174). After being washed three times with TBST, membranes were incubated at RT for 1 h with secondary antibody: Anti-Rabbit IgG (Goat), HRP-conjugated, Pre-absorbed (Nacalai Tesque, 21858). Immunoreactive bands were detected by enhanced chemiluminescence using Chemi-Lumi One Super (Nacalai Tesque, 2230). The intensities of the bands were densitometrically quantified using ImageJ.

R-ChIP and DNA recovery

R-ChIP was performed based on the published framework (Chen et al., 2019) with the experimental conditions described below. For each immunoprecipitation, 5.0×10^6 AF22 cells were harvested and cross-linked with 1% (v/v) formaldehyde for 10 min at room temperature, followed by quenching with glycine (final 125 mM) for 10 min. Cells were pelleted and lysed in SDS lysis buffer. Protease inhibitor cocktail (Nacalai Tesque, 25955-11) and recombinant RNase inhibitor (Takara, 2313B) were added to the SDS lysis buffer prior to sonication. Chromatin was fragmented by sonication on a Branson Sonifier 450 (output setting 2, continuous mode) using 13×6 s pulses with 1 min cooling on ice between pulses. After sonication, lysates were clarified by centrifugation (8°C, 15,000 rpm, 10 min) and diluted with ice-cold ChIP dilution buffer to a final volume of 2.0 mL. From the diluted chromatin, a 650 μL aliquot was supplemented with protease inhibitor cocktail (6.5 μL) and recombinant RNase inhibitor (8.125 μL), and subsequently split into immunoprecipitation (500 μL) and input (150 μL) aliquots.

For immunoprecipitation, 20 μL per sample of Dynabeads M-280 Sheep anti-Rabbit IgG (Invitrogen, Thermo Fisher Scientific, 11204D) were washed with 0.5% (w/v) globulin-free BSA (Nacalai Tesque, 01281-84) in PBS and pre-blocked at 4°C with rotation. Anti-V5 antibody (3 μL per sample; V5-Tag (D3H8Q) Rabbit Monoclonal Antibody; Cell Signaling Technology, #13202) was incubated with the beads at 4°C overnight with rotation. Chromatin was pre-cleared with blocked beads lacking antibody at 4°C for 1 h and then incubated with the antibody-conjugated beads at 4°C for 12–16 h with rotation. Input DNA was retained from the same chromatin preparation and processed in parallel.

Beads were washed at 4°C with rotation (5 min per wash) in the following order: 1× RIPA buffer containing 150 mM NaCl (three washes), 1× RIPA buffer containing 500 mM NaCl (three washes), Buffer III (LiCl wash buffer containing 0.5% non-ionic detergent and 0.5% sodium deoxycholate; three washes), and finally 1× TE (two washes). Wash buffers were supplemented with the protease inhibitor cocktail (Nacalai Tesque, 25955-11) and recombinant RNase inhibitor (Takara, 2313B) at 1:1000 (v/v). Elution buffer (200 μL) was supplemented with protease inhibitor cocktail (2 μL) and recombinant RNase inhibitor (2 μL) immediately prior to use. Immunoprecipitated material was eluted in the supplemented elution buffer at 65°C for 15 min. Input samples were adjusted to the same final volume with the supplemented elution buffer and treated identically thereafter. Eluates were digested with Proteinase K at 37°C for 6 h and reverse cross-linked at 65°C overnight, followed by RNase A treatment (final 20 μg/mL) at 37°C for 30 min. DNA was purified by phenol:chloroform:isoamyl alcohol extraction, precipitated with sodium acetate and isopropanol in the presence of glycogen as a carrier, washed with 70% ethanol, air-dried, and resuspended in TE (15 μL).

QUANTIFICATION AND STATISTICAL ANALYSIS

Experiments were conducted using at least three biological replicates for each group. Statistical analyses were performed by Student's t test (for two-group comparisons), and one-way ANOVA, followed by Tukey's multiple comparison tests, as appropriate (for multiple groups comparisons). Exact values of n (sample size) are provided in the figures and figure legends. For cell counting, three or more different areas were chosen in each sample of any group to avoid biased measurement. All data are presented as mean ± standard deviation (SD). Statistical significance was set at p-value less than 0.05.

Stem Cell Reports, Volume *21*

Supplemental Information

A human-specific non-coding RNA for *EFHC1*, an epilepsy-associated gene, regulates neural stem cell proliferation for cortical development

Sota Nishida, Boyang An, Hayato Uchida, Shota Zenno, Takumi Nojima, Arisa Makimura, Fumihiro Morishita, Mizuki Honda, and Takuya Imamura

Fig.S1

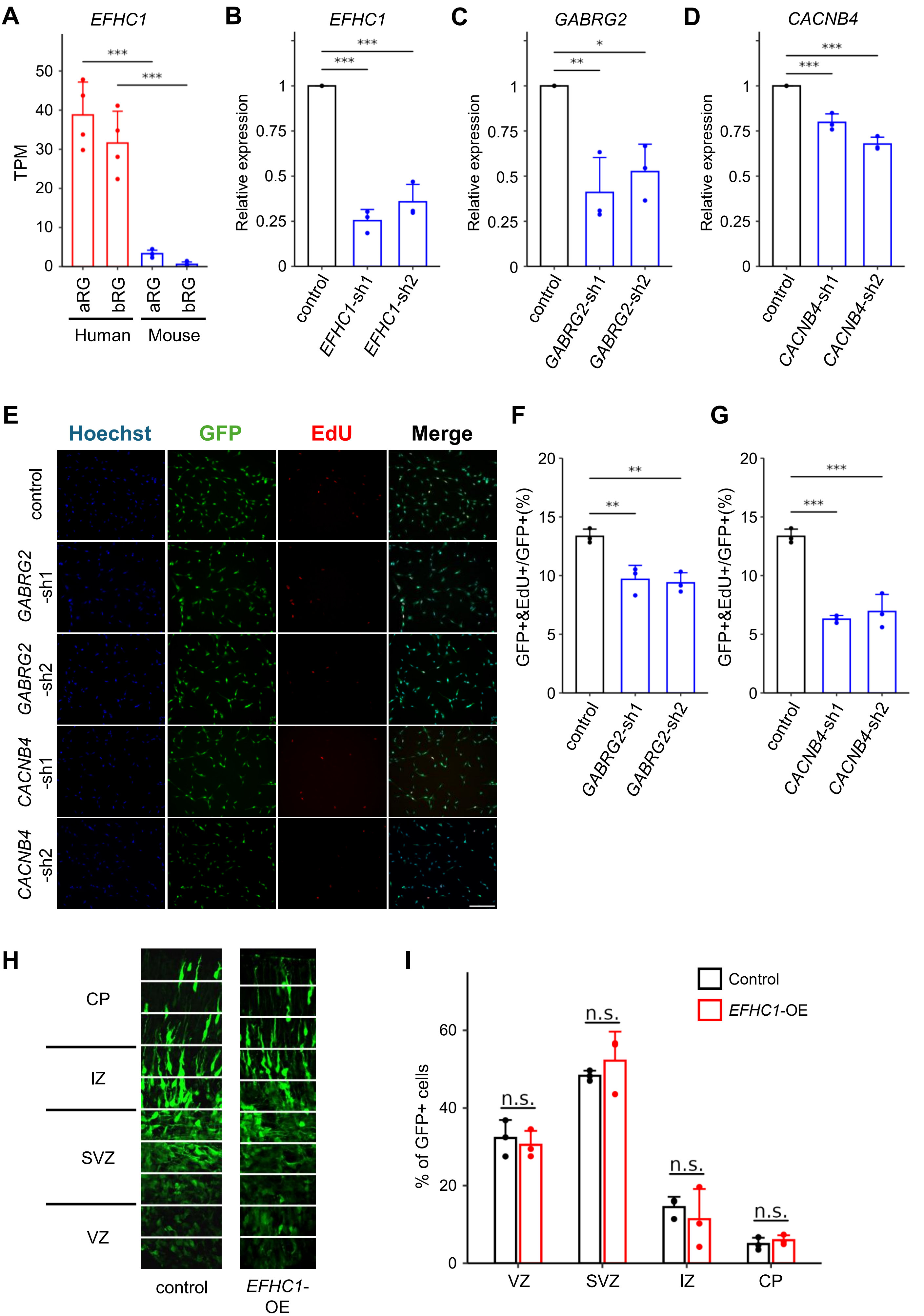

A
EFHC1
TPM
50
40
30
20
10
0
***
***
aRG
bRG
aRG
bRG
Human
Mouse
B
EFHC1
Relative expression
1
0.75
0.5
0.25
0
***
***
control
EFHC1-sh1
EFHC1-sh2
C
GABRG2
Relative expression
*
**
control
GABRG2-sh1
GABRG2-sh2
D
CACNB4
Relative expression
***
***
control
CACNB4-sh1
CACNB4-sh2
E
Hoechst
GFP
EdU
Merge
control
GABRG2 -sh1
GABRG2 -sh2
CACNB4 -sh1
CACNB4 -sh2
F
GFP+&EdU+/GFP+(%)
20
15
10
5
0
**
**
control
GABRG2-sh1
GABRG2-sh2
G
GFP+&EdU+/GFP+(%)
***
***
control
CACNB4-sh1
CACNB4-sh2
H
CP
IZ
SVZ
VZ
control
EFHC1-OE
I
Control
EFHC1-OE
% of GFP+ cells
60
40
20
0
n.s.
n.s.
n.s.
n.s.
VZ
SVZ
IZ
CP

Fig. S1

JME-associated genes that are highly expressed in humans play a role in NSC proliferation.

(A) The expression levels of *EFHC1* in aRG and bRG of humans and mice. $n = 4$ independent experiments; Tukey's test.

(B-D) Quantification of the mRNA level of *EFHC1* (B), *GABRG2* (C), and *CACNB4* (D) following knockdown. $n = 3$ independent experiments; Tukey's test.

(E) Representative images of human NSCs stained for GFP (green) and EdU (red) in control, *GABRG2* knockdown, and *CACNB4* knockdown. Nuclei were stained with Hoechst (blue).

(F and G) Quantification of EdU+ GFP+ cells to GFP+ cells under *GABRG2* knockdown (F) and *CACNB4* knockdown (G) as shown in (E). $n = 3$ independent experiments; Tukey's test.

(H) Representative images of the cortical slices stained for GFP (green) in control and *EFHC1*-overexpressing E15.5 developing mice. These images show the spatial distribution of GFP-positive cells across cortical developmental zones.

(I) Quantification of the ratio of GFP+ cells in the VZ, SVZ, IZ, and CP, respectively. n = 3 independent experiments; Tukey's test.

mean ± SD; $*P < 0.05$, $**P < 0.01$, $***P < 0.001$; scale bar = 100 μm.

Fig.S2

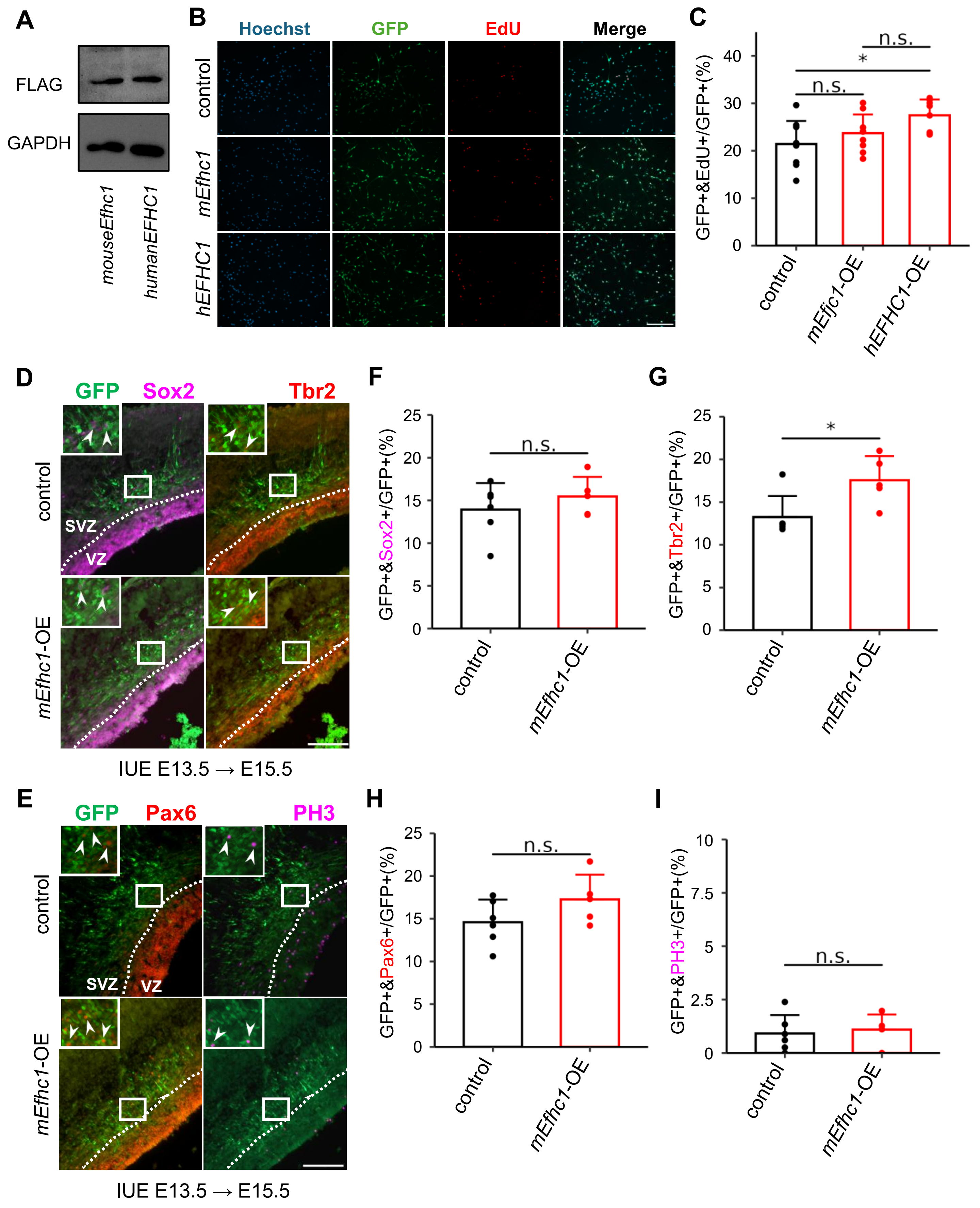
A
FLAG
GAPDH
mouseEfhc1
humanEFHC1
B
Hoechst
GFP
EdU
Merge
control
mEfhc1
hEFHC1
C
GFP+&EdU+/GFP+(%)
n.s.
*
n.s.
control
mEfjc1-OE
hEFHC1-OE
D
GFP
Sox2
Tbr2
control
mEfhc1-OE
SVZ
VZ
IUE E13.5 → E15.5
F
GFP+&Sox2+/GFP+(%)
n.s.
control
mEfhc1-OE
G
GFP+&Tbr2+/GFP+(%)
*
control
mEfhc1-OE
E
GFP
Pax6
PH3
control
mEfhc1-OE
SVZ
VZ
IUE E13.5 → E15.5
H
GFP+&Pax6+/GFP+(%)
n.s.
control
mEfhc1-OE
I
GFP+&PH3+/GFP+(%)
n.s.
control
mEfhc1-OE

Fig. S2

Mouse *Efhc1* expression slightly promotes NSC proliferation.

(A) Western blot analysis of whole cell lysates from mouse NSCs overexpressing either *mouseEfhc1* or *humanEFHC1*. The expression levels of *mouseEfhc1* and *humanEFHC1* were compared using the FLAG tag present in both vectors.

(B) Representative images of E14.5 mouse NSCs stained for GFP (green) and EdU (red) in control, human *EFHC1* overexpression, and mouse *Efhc1* overexpression. Nuclei were stained with Hoechst (blue).

(C) Quantification of the ratio of EdU+ GFP+ cells to GFP+ cells shown in (A). $n = 9$ (control and *mEfhc1*-OE) and 8 (*hEFHC1*-OE) independent experiments; Tukey's test.

(D) Representative images of the cortical slices stained for GFP (green), Sox2 (magenta), and Tbr2 (red) in control and mouse *Efhc1*-overexpressing E15.5 developing mice. Nuclei were stained with Hoechst (blue).

(E) Representative images of the cortical slices stained for GFP (green), Pax6 (red), and PH3 (magenta) in control and mouse *Efhc1*-overexpressing E15.5 developing mice. Nuclei were stained with Hoechst (blue).

(F-I) Quantification of the ratio of Sox2+ (E), Tbr2+ (F), Pax6+ (G), and PH3+ (H) GFP+ cells to GFP+ cells in SVZ. $n = 6$ (control) and 5 (*mEfhc1*-OE) independent experiments; Student's *t* test.

IUE: *in utero* electroporation; OE: overexpression; mean ± SD; $^*P < 0.05$, n.s.: not significant; scale bar = 100 μm.

Fig.S3

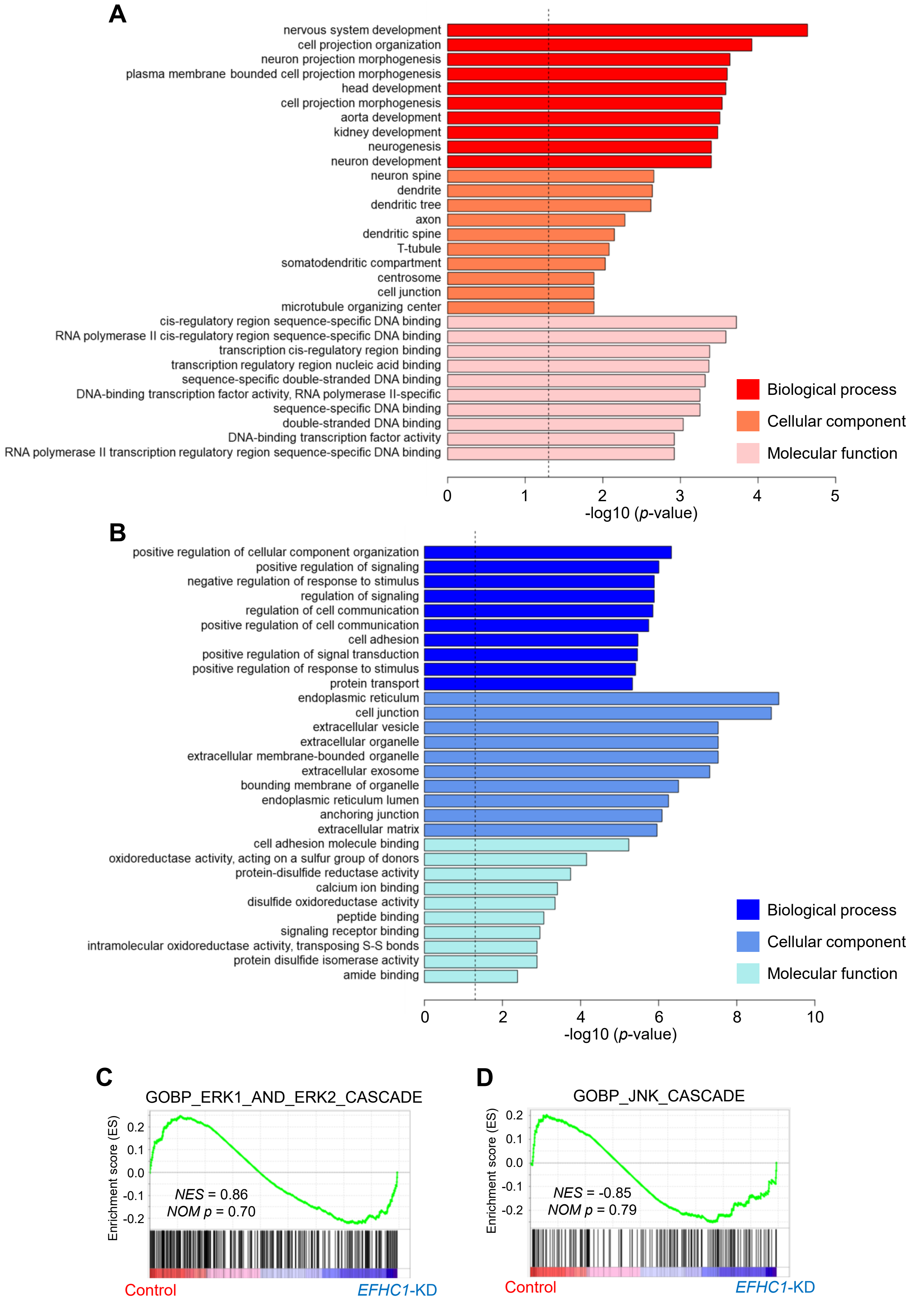
A
nervous system development
cell projection organization
neuron projection morphogenesis
plasma membrane bounded cell projection morphogenesis
head development
cell projection morphogenesis
aorta development
kidney development
neurogenesis
neuron development
neuron spine
dendrite
dendritic tree
axon
dendritic spine
T-tubule
somatodendritic compartment
centrosome
cell junction
microtubule organizing center
cis-regulatory region sequence-specific DNA binding
RNA polymerase II cis-regulatory region sequence-specific DNA binding
transcription cis-regulatory region binding
transcription regulatory region nucleic acid binding
sequence-specific double-stranded DNA binding
DNA-binding transcription factor activity, RNA polymerase II-specific
sequence-specific DNA binding
double-stranded DNA binding
DNA-binding transcription factor activity
RNA polymerase II transcription regulatory region sequence-specific DNA binding
Biological process
Cellular component
Molecular function
0
1
2
3
4
5
-log10 (p-value)
B
positive regulation of cellular component organization
positive regulation of signaling
negative regulation of response to stimulus
regulation of signaling
regulation of cell communication
positive regulation of cell communication
cell adhesion
positive regulation of signal transduction
positive regulation of response to stimulus
protein transport
endoplasmic reticulum
cell junction
extracellular vesicle
extracellular organelle
extracellular membrane-bounded organelle
extracellular exosome
bounding membrane of organelle
endoplasmic reticulum lumen
anchoring junction
extracellular matrix
cell adhesion molecule binding
oxidoreductase activity, acting on a sulfur group of donors
protein-disulfide reductase activity
calcium ion binding
disulfide oxidoreductase activity
peptide binding
signaling receptor binding
intramolecular oxidoreductase activity, transposing S-S bonds
protein disulfide isomerase activity
amide binding
Biological process
Cellular component
Molecular function
0
2
4
6
8
10
-log10 (p-value)
C
GOBP_ERK1_AND_ERK2_CASCADE
Enrichment score (ES)
0.2
0.1
0.0
-0.1
-0.2
NES = 0.86
NOM p = 0.70
Control
EFHC1-KD
D
GOBP_JNK_CASCADE
Enrichment score (ES)
0.2
0.1
0.0
-0.1
-0.2
NES = -0.85
NOM p = 0.79
Control
EFHC1-KD

Fig. S3

***EFHC1* knockdown alters NSC pools, providing more premature neurons, and is enriched in the endoplasmic reticulum.**

(A and B) GO enrichment analysis of DEGs in *EFHC1* knockdown human NSCs. The top 10 enriched GO terms for upregulated (A) and for downregulated (B) DEGs are shown. Gene ontology categories included biological process, cellular component, and molecular function. Dotted lines indicate significance ($p < 0.05$).

(C and D) GSEA of *EFHC1* knockdown AF22 cells for Erk1 and Erk2 cascade (C) and Jnk cascade (D). NES: normalized enrichment score; NOM p: nominal *p*-value.

Fig.S4

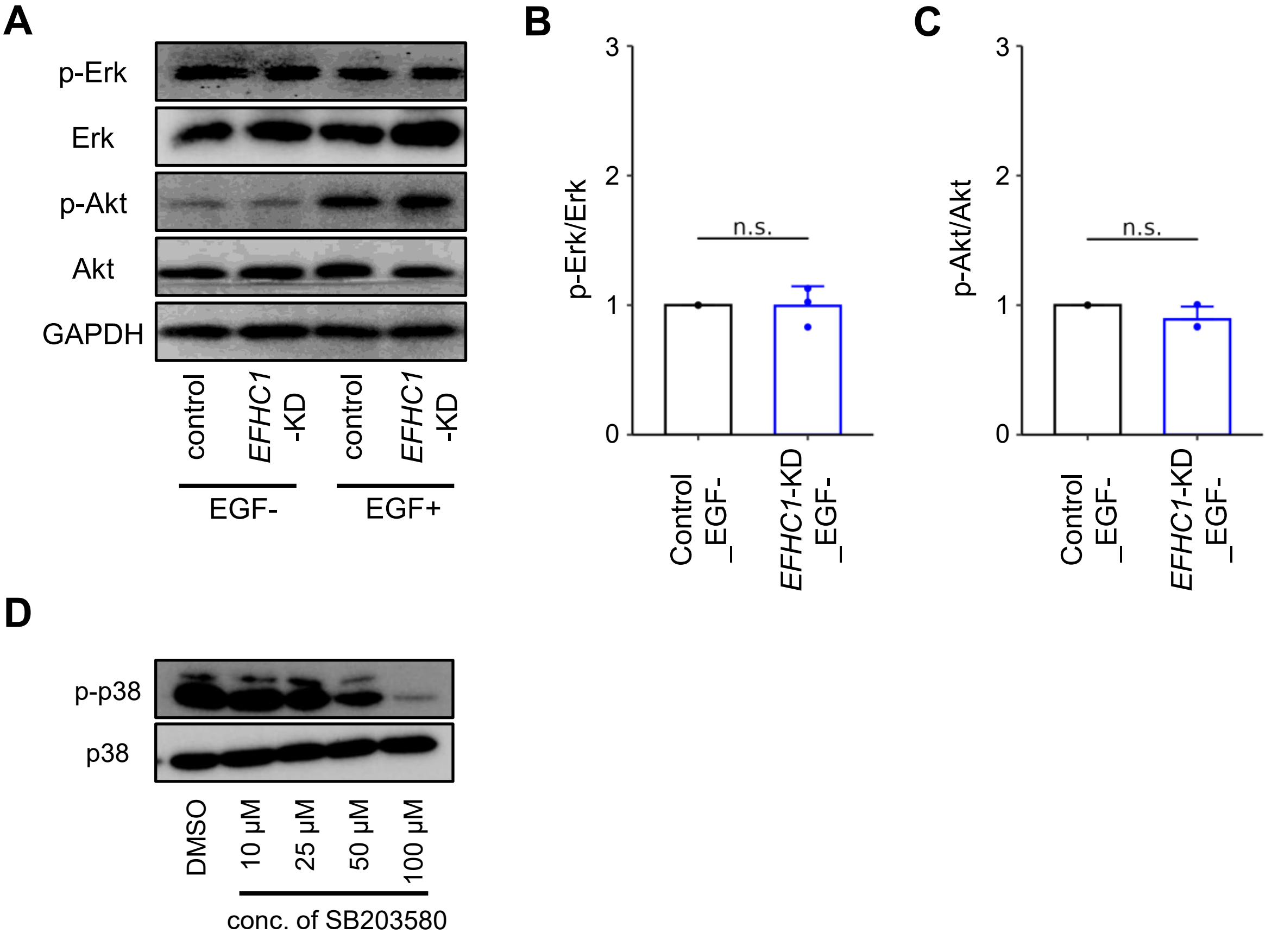
A
p-Erk
Erk
p-Akt
Akt
GAPDH
control
EFHC1 -KD
control
EFHC1 -KD
EGF-
EGF+
B
p-Erk/Erk
n.s.
3
2
1
0
Control _EGF-
EFHC1-KD _EGF-
C
p-Akt/Akt
n.s.
3
2
1
0
Control _EGF-
EFHC1-KD _EGF-
D
p-p38
p38
DMSO
10 μM
25 μM
50 μM
100 μM
conc. of SB203580

Fig. S4

***EFHC1* does not affect the activation of Erk or Akt.**

(A) Western blot analysis of whole cell lysates from growth factor-starved control or *EFHC1* knockdown human NSCs treated with EGF 5 min. p-Erk: phospho-Erk, p-Akt: phospho-Akt.

(B and C) Quantification of Erk (B) and Akt (C) phosphorylation levels without EGF in western blot analysis. The values were normalized to control band intensity. n = 3 independent experiments; Student's t test.

(D) Western blot analysis of whole cell lysates from *EFHC1* knockdown human NSCs treated with SB203580 dissolved by DMSO. p-p38: phospho-p38; conc.: concentration.

Fig.S5

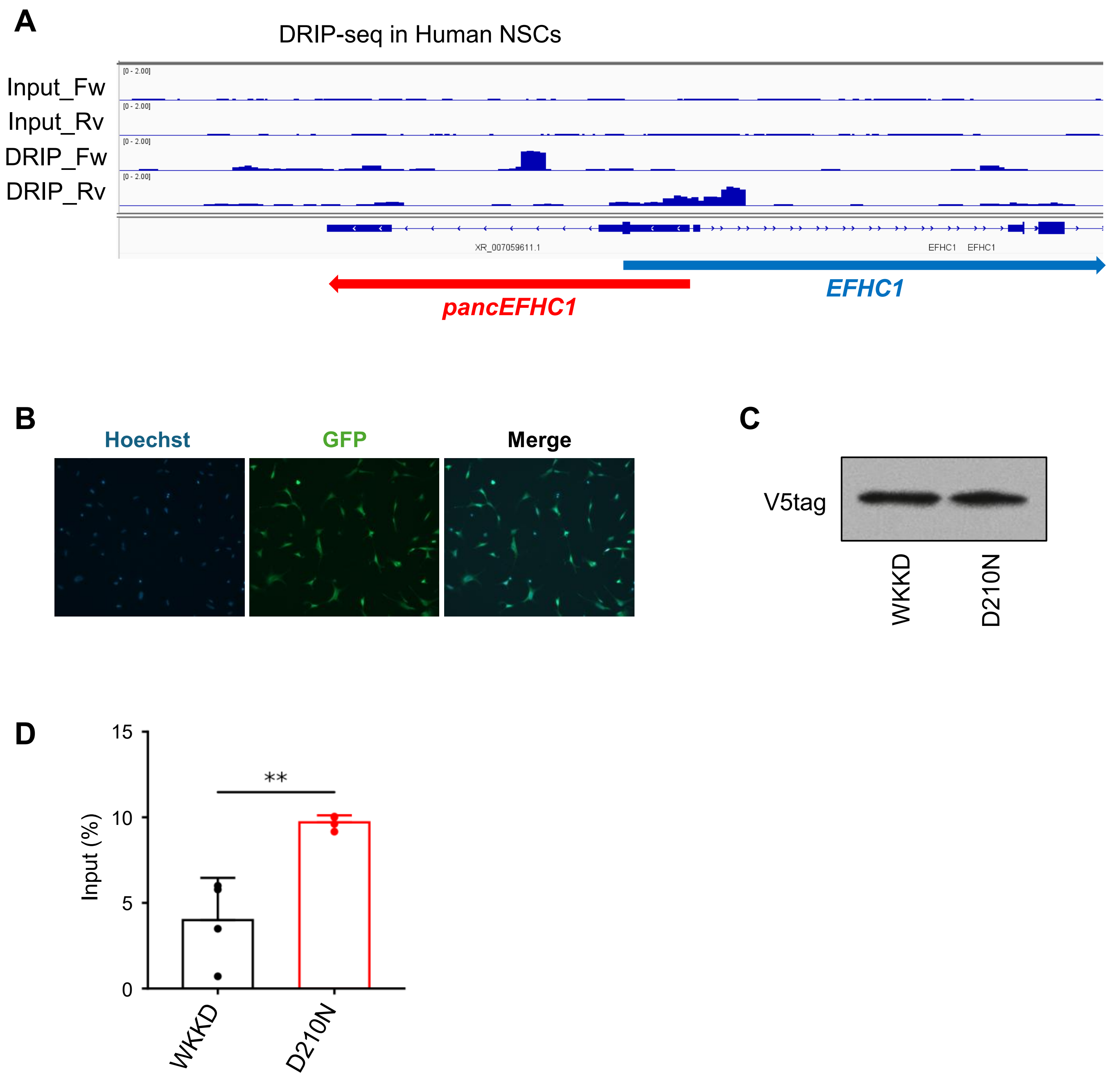
A
DRIP-seq in Human NSCs
Input_Fw
Input_Rv
DRIP_Fw
DRIP_Rv
XR_007059611.1
EFHC1
EFHC1
pancEFHC1
EFHC1
B
Hoechst
GFP
Merge
C
V5tag
WKKD
D210N
D
Input (%)
15
10
5
0
**
WKKD
D210N

Fig. S5

***pancEFHC1* forms an R-loop structure.**

(A) DRIP-seq analysis of forward and reverse strands in human NSCs, showing R-loop peak signals. Blue and red arrows indicate the transcriptional directions of *EFHC1* and *pancEFHC1*, respectively.

(B) Representative images of human NSCs overexpressing RNase H1 mutants and stained for GFP (green), showing nearly 100% infection efficiency. Nuclei were stained with Hoechst (blue).

(C) Western blot analysis of whole cell lysates from human NSCs overexpressing either WKKD or D210N. The expression levels of WKKD and D210N were compared using the V5 tag present in both vectors.

(D) qPCR analysis of purified V5-immunoprecipitated DNA to quantify enrichment at the *EFHC1* locus, shown as percent input). $n = 3$ independent experiments; Student's t test.

mean ± SD; $^{**}P < 0.01$.

Fig.S6

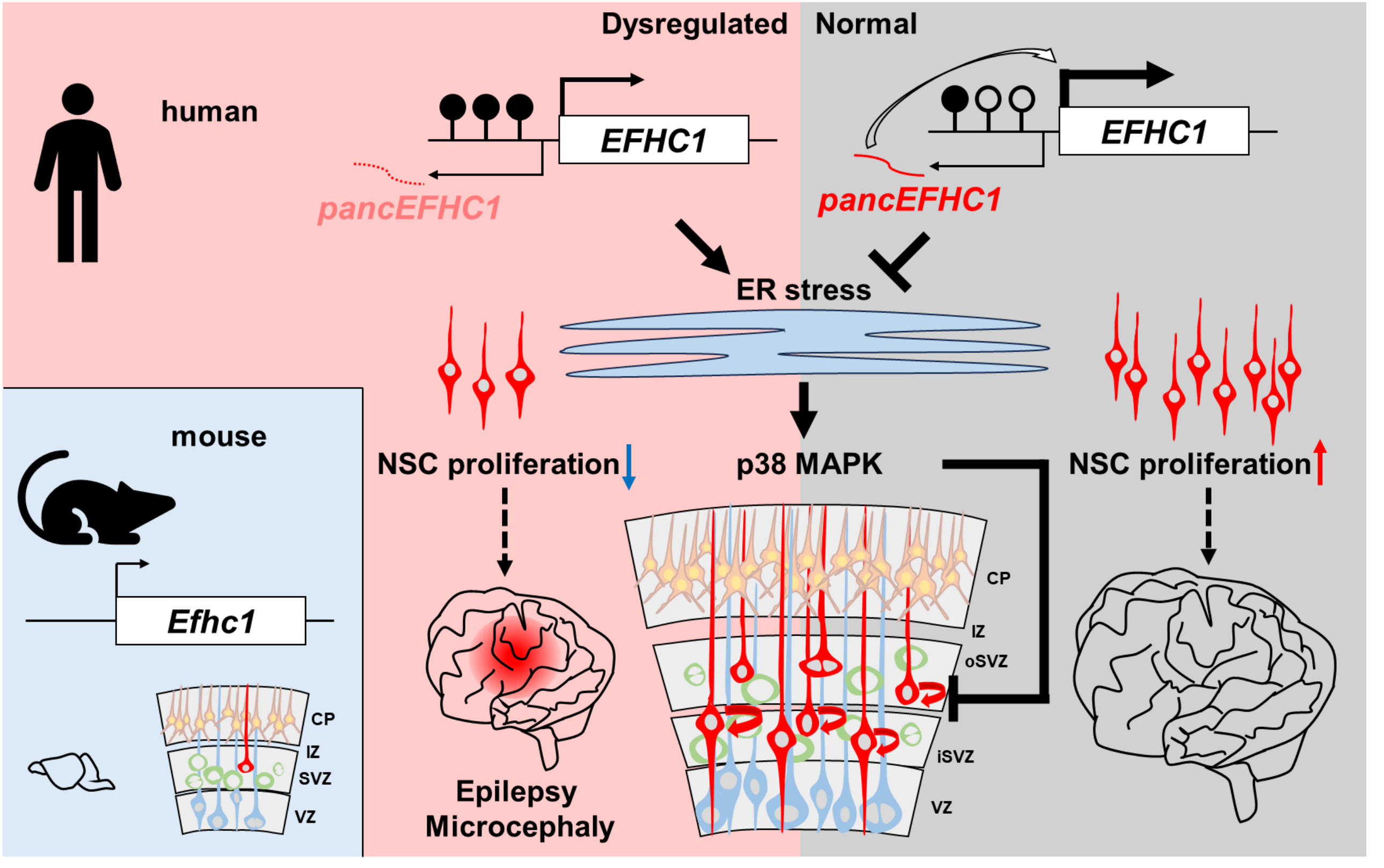

Dysregulated
Normal
human
EFHC1
pancEFHC1
EFHC1
pancEFHC1
ER stress
mouse
NSC proliferation
p38 MAPK
NSC proliferation
Efhc1
CP
IZ
oSVZ
iSVZ
VZ
CP
IZ
SVZ
VZ
Epilepsy
Microcephaly

Fig. S6

Schematic illustration depicting the effects of *pancEFHC1*/*EFHC1* on NSC proliferation and cortical development.
